# Unraveling the chaotic genomic landscape of primary and metastatic canine appendicular osteosarcoma with current sequencing technologies and bioinformatic approaches

**DOI:** 10.1371/journal.pone.0246443

**Published:** 2021-02-08

**Authors:** Shirley Chu, Zachary L. Skidmore, Jason Kunisaki, Jason R. Walker, Malachi Griffith, Obi L. Griffith, Jeffrey N. Bryan

**Affiliations:** 1 Department of Veterinary Medicine and Surgery, University of Missouri, Columbia, MO, United States of America; 2 McDonnell Genome Institute, Washington University, St. Louis, MO, United States of America; 3 Department of Medicine, Washington University, St. Louis, MO, United States of America; Colorado State University, UNITED STATES

## Abstract

Osteosarcoma is a rare disease in children but is one of the most common cancers in adult large breed dogs. The mutational landscape of both the primary and pulmonary metastatic tumor in two dogs with appendicular osteosarcoma (OSA) was comprehensively evaluated using an automated whole genome sequencing, exome and RNA-seq pipeline that was adapted for this study for use in dogs. Chromosomal lesions were the most common type of mutation. The mutational landscape varied substantially between dogs but the lesions within the same patient were similar. Copy number neutral loss of heterozygosity in mutant *TP53* was the most significant driver mutation and involved a large region in the middle of chromosome 5. Canine and human OSA is characterized by loss of cell cycle checkpoint integrity and DNA damage response pathways. Mutational profiling of individual patients with canine OSA would be recommended prior to targeted therapy, given the heterogeneity seen in our study and previous studies.

## 1. Introduction

Osteosarcoma (OSA) is a rare disease in humans, but it is the 8^th^ most common cancer in children, and 30% of affected children succumb to their cancer within 5 years [1]. On the other hand, OSA is estimated to occur 55 times more commonly in dogs at an incidence of 272 cases/million per year [2], compared to an incidence of 5 cases/million per year in humans aged 0–19 years old [3]. The incidence is expected to be even higher in large/giant breed dogs compared to the general dog population. Dogs are arguably the best animal model to study human OSA because of the many similarities [4–8]. Both cancers are histologically similar, metastatically aggressive, and are treated with therapies that include surgery and platinum-based chemotherapy [5]. Risk factors for both canine and human OSA are large body size [2, 9, 10] and radiation therapy exposure [11, 12]. OSA risk is thus hypothesized to increase with increased number of cell divisions and DNA damage respectively. Chromosomal features such as aneuploidy, copy number and structural variations, and genomic instability are also common in both species. *TP53* is mutated in 100% of human OSAs [13] and 71–83% of canine OSAs [14, 15]. Common pathways determined to be OSA driver pathways are also dysregulated in both species, including the Wnt and PI3K/mTOR pathways [16].

At least 63 human OSA primary tumors and 16 metastatic tumors with paired normal samples have been sequenced with whole genome sequencing (WGS), whole exome sequencing (WES), and RNA-seq (St. Jude PeCan database). In comparison a combined WGS, WES and RNA-seq approach has not been done in a canine OSA primary or canine OSA metastatic tumor to date to the authors’ knowledge [15]. Exome studies in canine OSA have been more frequently published, with 93 primary canine OSA and 10 metastatic canine OSA exomes sequenced to date [14, 15]. Given the complex karyotype and genetic heterogeneity of human and canine OSA [15], sequencing of a large number of canine patients will be required to classify mutations into drivers and passengers, as well as to identify subtypes of canine OSA that have different responses to treatments and outcomes.

WES is usually restricted to the protein coding regions, which occupy ~3% of the canine genome [17]. WES would be sufficient for identifying small-scale coding mutations (e.g. indels, single nucleotide variants or SNVs) and would be moderately effective but not ideal for copy number alterations or CNAs (poor resolution and higher false positive rates). WES would fail to capture the majority of the structural variants that make up the complex karyotype of OSA. WGS, WES and RNA-seq are complimentary methodologies and comprehensive sequencing with all three overcomes the shortfalls of each individual approach [18]. This is the first study to perform combined WGS, WES, and RNA-seq on both a primary and metastatic lesion from each of two dogs and thus provides the most in-depth genomic study of a canine OSA patient to date. This is also the first study to perform loss of heterozygosity (LOH) analysis in dogs with OSA.

The purposes of this study were to (1) add to the growing genomic knowledgebase for canine OSA and to describe new insights into the mutational landscape of canine OSA and how this compares to human OSA; (2) adapt an automated comprehensive human cancer informatics pipeline for the dog [19]; (3) identify the drivers of and characteristics of chromosomal instability in canine OSA; (4) compare the primary and metastatic lesions to establish a sequence of events of tumor evolution and (5) show proof of concept of a comprehensive genome-informed approach to identify actionable driver pathways for individual patients.

## 2. Results

### 2.1. Samples

Both patients in this study were adult large breed dogs, S1 Table. The overall survival time of both dogs was ~1.5 years which is longer than the median survival time of ~10 months seen in dogs with OSA treated with standard of care therapy consisting of amputation and carboplatin, S2 Table [20]. The longer survival in this study is likely due to the selection of dogs that had a pulmonary metastasectomy, typically only performed in the setting of oligometastasis. Sequencing metrics are presented in S3 and S4 Tables. Tumor purity (estimated as the *TP53* variant allele frequency) was too low in the metastatic lesion of the Labrador Retriever to make conclusions about the small and large scale mutations in this sample.

### 2.2. Single Nucleotide Variants (SNVs)

#### 2.2.1. TP53 was recurrently mutated via a missense mutation

Thirty-seven and twenty-eight SNVs passed manual review in the Labrador and Sheepdog sample respectively, S1 Fig and Table 1. The exonic SNV mutation rate was low at 0.1–0.2/Mb compared to the 1.38/Mb seen in a previous WES study [15]. This very low rate was likely due to the stringent manual review criteria used in this study [21] or to differences in the populations studied. Only, *TP53* was mutated in both dogs. WGS/exome/RNA data showed that the variant allele frequency (VAF) for *TP53* was 0%, 76.7–87.5% and 64.7–89.1% in the matched normal, primary and metastatic lesion respectively in the Sheepdog. Correspondingly in the Labrador, the VAF for *TP53* was 0%, 79.1–94.4% and 11.6–33.3%. The tumor purity and the prevalence of the *TP53* mutation in the tumor cell population was high in all samples except the metastatic sample in the Labrador

**Table 1:** Frequency of mutation type in each osteosarcoma sample.

	Sheepdog			Labrador Retriever		
	Primary	Metastatic	% shared	Primary	Metastatic	% shared
**SNV**	17	26	54%	34	22	51%
**Inversion**	20	22	50%	23	17	0%
**Translocation**	76	71	79%	42	16	38%
**Deletion**	25	33	57%	30	22	53%
**Duplication**	15	21	57%	19	7	53%
**CNA Loss**	5888	5822	61%	3475	12	0%
**CNA Gain**	2191	3155	43%	3019	286	9%
**Expressed gene fusion**	3	4	75%	3	N/A	0%

SNV = single nucleotide variant including indels in exons and splice sites; CNA = copy number alternation

CNAs were counted in the context of gene count.

% shared will be an underestimate in the Labrador due to low tumor purity in the metastatic lesion.

Structural variant counts and % shared will likely be an overestimate and underestimate respectively due to redundancies that result from discrepancies in SV calling in a large genome.

Genes with SNVs that were predicted to have a high impact on function included, *TNN* (primary and metastasis), *ARHGAP12* (primary), *SNX9* (primary), *SMARCA5* (primary), and *ATP8B4* (primary) in the Labrador and *GZMK* (metastasis), *ABCG4* (metastasis) and *DNAH14* (primary and metastasis) in the Sheepdog. Corresponding SNVs in the human orthologs for these genes were not found in the COSMIC database for any cancer (accessed 9-Jul-2019). *SMARCA5* was identified as a potential oncogenic driver in a mouse model of OSA [22]. *SNX9*, a scaffold protein involved in actin assembly and clathrin-mediated endocytosis, has been implicated in mitosis, invasion and metastasis [23]. Genes in the COSMIC cancer census database (version 89) with a SNV included, *TP53* and *MYO5A* in the Labrador and *TP53*, *NOTCH2*, *ZNF521* and *PICALM* in the Sheepdog. All of these SNVs were predicted to have a moderate impact on function. The *PICALM* SNV in the Sheepdog was a non-synonymous mutation in exon 9/21 (ENSCAFT00000044406.1). A *PICALM-DLG2* fusion was also detected in the primary and metastatic lesion of the Sheepdog, S2 Fig. The gene fusion co-occurred with deletion of *PICALM* with ~0.5–1.5 copies. A non-synonymous SNV, p.Asp456Tyr, was found in exon 8 of *NOTCH2* (ENSCAFT00000016889.4) of the Sheepdog metastatic lesion; this mutation has not been reported in the COSMIC database (accessed 9-Jul-2019) but has been reported in a metastatic canine OSA lesion [15]. *NOTCH2* was also amplified in the Sheepdog with the *NOTCH2* SNV with ~3 copies in the primary and metastatic lesion. *NOTCH2* was also a highly expressed gene with fragments per kilobase of exon model per million reads mapped (FPKMs) of 40–84 in all evaluable samples.

#### 2.2.2. Strong correlation between SNV profile of the primary and metastatic lesion

**Fig 1:**
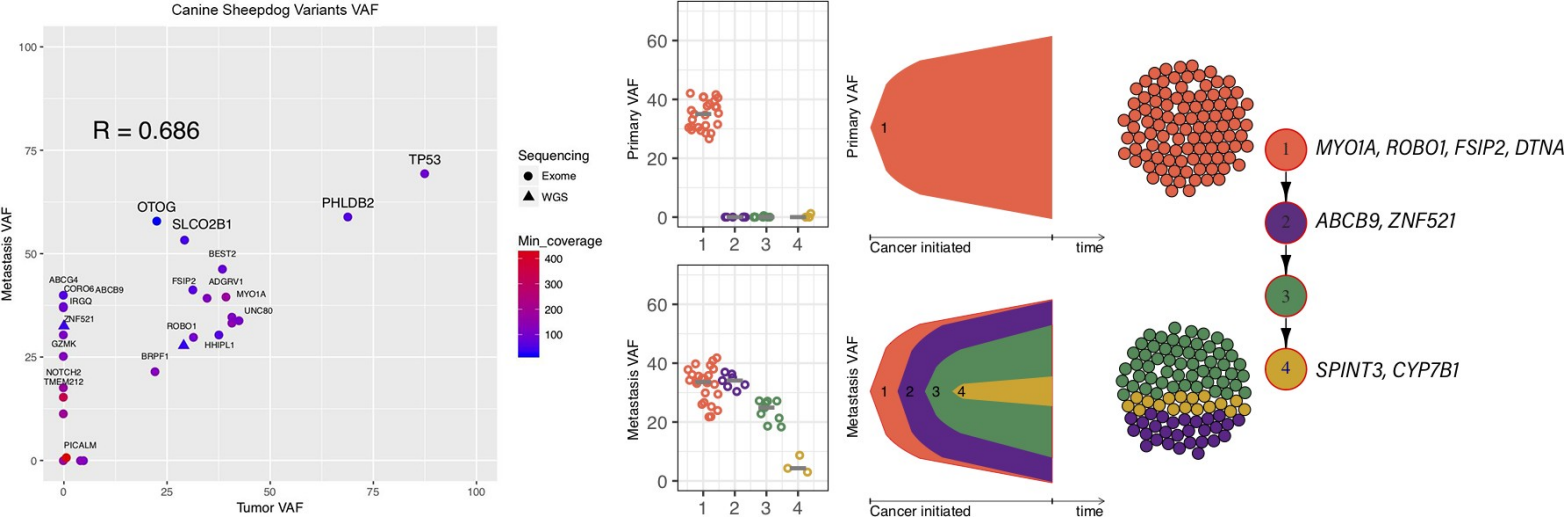
A metastasis specific clone is identified upon comparison of the SNV profiles in the primary and metastatic lesion. a. Strong correlation between SNVs seen in the primary compared to the metastatic lesion in the Sheepdog. VAF, variant allele frequency. If the variant was found in the WGS and exome data the highest VAF was reported. Genes without a gene symbol were not labeled. Note the presence of metastatic specific variants (left side of the figure). Non-synonymous SNVs are not shown. b. Clonal evolution analysis revealed that the primary clone was also found in the metastatic lesion but was at a lower frequency than the metastatic specific clones. The exonic synonymous and non-synonymous SNVs that characterize these clones are labelled. 1: primary clone; orange. 2–4: metastatic clones; purple, green and yellow. There were no exonic SNVs that were unique to clone 3. The estimate of the percentage of cells in the metastatic lesion that were consistent with clone 1 was −9.4% to 5.7% (p = 0.740), clone 2 was 22.0% to 38.7% (p = 0.000), clone 3 was 46.6% to 66.3% (p = 0.000) and clone 4 was 10.4% to 22.0% (p = 0.000).

Low tumor purity in the metastatic lesion of the Labrador precluded accurate comparisons of the SNV landscape between the primary and metastatic lesion for this patient, S3 Fig. However, in the Sheepdog there was strong correlation between the SNVs in the primary and metastatic lesions, as well as evidence of clonal heterogeneity, Fig 1A. Of the 28 SNVs, 2 and 11 of the SNVs were unique to the primary and metastatic lesion respectively, S5 Table. Metastasis-specific SNVs were found in *ABCB9*, *ABCG4*, *CORO6*, *IRGQ*, *GZMK*, *NOTCH2*, *OR2T35*, *TMEM212*, *ZNF521*, *LOC102152223* and *ENSCAFG00000029779*. *ABCB9* and *ABCG4* are ATP-binding cassette membrane proteins in the multidrug resistant and White subfamilies respectively and the VAF was almost 50% in both genes in the metastatic lesion. Clonal analysis revealed that the primary lesion was composed of one main clone and the metastatic lesion was composed of 4 main clones, Figs 1B and S4 and S6 Table. The clones in the metastatic lesion had all of the SNVs that were found in the primary clone but with additional SNVs. The predominant metastatic clone did not have a unique non-synonymous exonic SNV but was suspected to have a chromosomal mutation that allowed it to have a competitive advantage in the metastatic site. This metastatic clone was not found in the primary lesion by ClonEvol.

#### 2.2.3. Genome wide SNV mutational patterns

Kataegis is a phenomenon characterized by localized hypermutation with C→T and C→G substitutions at TpCpX sites which most closely resembles published genome wide mutational signatures 2 and 13 [24]. Kataegis was not seen in the samples in this study but was seen in 50% of pediatric OSAs [13] and in 16% (4/24) of canine OSAs [14], S5 Fig. Mutational signature 1 was the most common mutation signature seen in all samples. Signature 17 was seen in the primary and metastatic lesions in the Labrador and the metastatic lesion of the Sheepdog. Signatures 9 and 15 were seen in the primary and metastatic lesions in the Sheepdog. Signatures, 6, 8, 9, 24 and 25 were seen in one lesion, S6 Fig. Signature 1 is the most common signature in cancers in general, including pediatric and canine osteosarcoma. Signatures 9 and 17 have been previously reported in canine osteosarcoma [14, 15].

### 2.3. Chromosomal lesions

**Fig 2:**
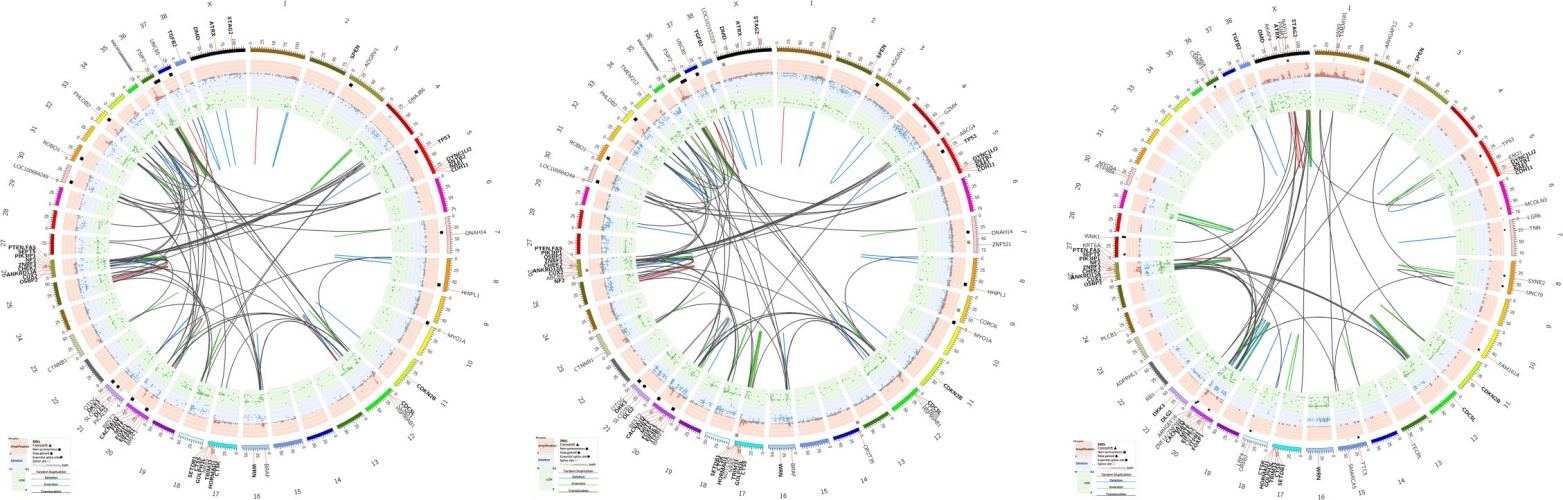
Large scale mutations were more common than small scale mutations. SNVs, LOH, CNAs, and SVs are shown. Gene labels are shown for all SNVs with a gene name and selected genes with other types of mutations. Genes in bold indicate genes that were commonly mutated in both patients. SVs are depicted in the innermost circle. a. Sheepdog–primary. b. Sheepdog–metastatic. c. Labrador—primary and metastatic lesions (combined due to the low tumor purity in the metastatic lesion).

A summary of the mutations identified and gene expression in both dogs can be seen in Table 1, S5, S7–S9 Tables and Fig 2A–2C. The Labrador had a more normal karyotype than the Sheepdog. Genes were most frequently mutated via a deletion. A comparison of the genes that were mutated via an amplification or deletion in each lesion can be seen in S7A and S7B Fig.

Chromosome 26 was the most severely affected chromosome in both dogs, S8A and S8B Fig. None of the amplified genes on chromosome 26 were in the COSMIC database. COSMIC tumor suppressor genes (TSGs) that were deleted and had low expression, FPKMs of 0–4, on chromosome 26 included *FAS*, *SEPT5* and *LZTR1* in all lesions examinable and *CHEK2*, *ZNRF3*, *NF2* and *CLTCL1* in both primary lesions. *PTEN* was deleted and moderately expressed in all lesions. In the dog these TSGs are clustered in 3 chromosomal regions on chromosome 26. The 3 regions contain the following adjacent genes, (1) *CHEK2*, *ZNRF3* and *NF2*; (2) *SEPT5*, *CLTCL1* and *LZTR1;* and lastly (3) *PTEN* and *FAS*. The distances between the genes in each region are conserved with humans. The major difference in dogs compared to humans is that in humans the region containing *PTEN* and *FAS* (chr10) is on a different chromosome than the first 2 regions (chr22). The chromosomal partner for translocations involving chromosome 26 in the Sheepdog was chromosome 4 but there were no translocations between chromosomes 4 and 26 in the Labrador. The pattern of SVs was complex and suggests chromoanagenesis, or chromosomal regeneration. Chromoanagenesis can produce a vast number of mutations in a single event and can lead to rapid tumor evolution. In the current study more specifically, chromothripsis or chromosome shattering resulting from a single event and repair via non-homologous end joining (NHEJ) was suggested by the genes that were reassembled in random order and orientation on chromosome 26 with resulting loss of chromosomal segments. Chromothripsis is estimated to occur in 20–25% human OSAs [13, 25]. The replication-based process, chromoanasynthesis was suggested by the clusters of gene amplifications and deletions that occurred. Chains of translocations with frequent deletions at the breakpoints that are characteristic of chromoplexy were also seen in the Sheepdog lesions. In addition to chromosome 26, chromosomes 12 and 20 were the most common chromosomes to have SVs in both dogs. Differences in SV distribution between the dogs included, a predilection for SVs on chromosome X in the Labrador and chromosomes 17, 25, 34 and 36 in the Sheepdog. Although similar genes were mutated via SVs in both dogs, the translocation partners were different in the two dogs and none of the translocations on any chromosome were recurrent in the 2 dogs.

#### 2.3.1. Copy number alterations and loss of heterozygosity were common

In both dogs, there were large segments of chromosomes that were not heterozygous in the germline. This was expected on chromosome X since both dogs were male. On the autosomes this was attributed to loci that have limited variability within a breed or individual due to inbreeding. Breed genetics may thus limit thorough somatic LOH analysis in purebred dogs. This was especially evident in the Sheepdog, S9 Fig.

Low tumor purity in the metastatic lesion of the Labrador precluded accurate CNA and LOH analysis of this sample. Percent aneuploidy in the genome was 20–27%, which was similar to the estimates for canine glioma, S11 Table. A higher percentage of the genome was affected by losses compared to gains, 12–19% compared to 5–9%. In both dogs, amplifications were numerous and always focal with the exception of chromosome X in the Labrador (0–74 Mb). In both dogs, whole chromosomal deletions of chromosomes 16 and 21 and segmental deletions of chromosomes 20, 26, 37 and 38 (0–13.5 Mb) were common, S10 Fig. Chromosomes 14 and 31 were the least affected by CNAs in both primary tumor samples and the Sheepdog metastatic sample, S11A and S11B Fig.

**Fig 3:**
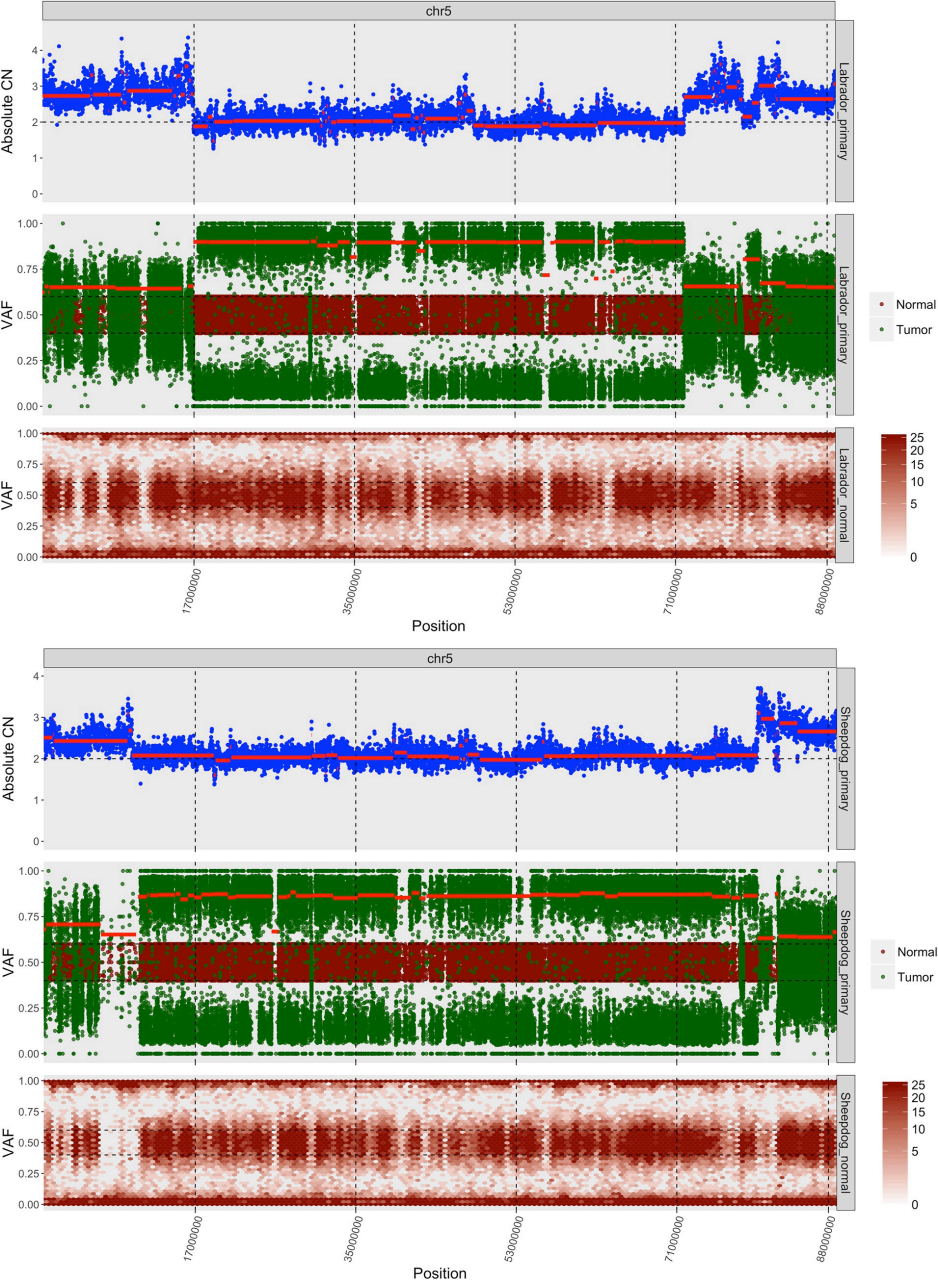
(a and b): Segmental copy number neutral loss of heterozygosity (CN-LOH) in the middle of chromosome 5 is shown in both dogs. *TP53* was located in this segmental region of CN-LOH. Representative CNA (top panel), somatic LOH (middle panel) and germline LOH (bottom panel) plots from the primary lesions in the Labrador and Sheepdog. The CN-LOH pattern of the metastatic lesion of the Sheepdog was similar to the primary and is not shown. (a) Labrador. (b) Sheepdog.

Somatic LOH patterns were shared in both patients on chromosomes 5, 12, 16, 20, 26, 37 and 38, S12 Fig and S12 Table. LOH patterns were very similar in the primary and metastatic lesion of the Sheepdog with the exception of an additional large deletion and resulting LOH on chromosome 2 in the metastatic lesion. Copy-number neutral LOH was seen in a 17–72 Mb region in the middle of chromosome 5 in all evaluable samples. *TP53* is located in this region, Fig 3A and 3B. A loss/duplication event in the *TP53* locus likely occurred after the missense mutation in *TP53*. The copy-number neutral LOH likely occurred due to mitotic recombination or duplication following unsuccessful repair of a dsDNA break [26]. Functionally, both patients were *TP53*^*mut/mut*^.

#### 2.3.2. Key genes in the PI3K/Akt and RB1 pathways were dysregulated

*PTEN* is located on the chromosome with the most chromosomal lesions, chromosome 26. Deletion was noted in all evaluable samples, with ~0.2 copies in the primary tumor from the Labrador and ~1.4 and 0.7 copies in the primary and metastatic lesion of the Sheepdog respectively, S13A and S13B Fig. CNAs were not detected in *PIK3CA*, *MTOR* or *AKT1* but an *AKT2* amplification was seen in the primary lesion in the Sheepdog and *PIK3CB* amplification was seen in the Labrador lesions and the Sheepdog metastatic lesions. LOH and focal deletion of *CDKN2B* were seen in both dogs resulting in ~0.5 copies, S14A and S14B Fig. The deleted locus in the Sheepdog also included *CDKN2A*. CNAs were not noted in *CDK4*, which is inhibited by *CDKN2A/B*, but was highly expressed with FPKMs of 36–182 in all evaluable samples, especially the metastatic lesion in the Sheepdog. *RB1* was deleted in the primary lesion of the Labrador via a chromosomal deletion resulting in ~1.2 copies and consequently *RB1* expression was lower in the Labrador.

#### 2.3.3. Somatic deletions of genes that predispose human patients to hereditary cancer risk syndromes were common

Germline losses of *TP53*, *WRN*, *RB1*, *RECQL4* and *BLM* are risk factors for hereditary OSA in people. Somatic loss of *TP53* (via SNV) and *WRN* (via deletion) were seen in all samples in this study. There were ~1–1.5 copies of *WRN* with LOH. Somatic deletion of *RB1* was seen in the Labrador only. *NF2* was deleted in both primary tumors with ~1–1.5 copies. Somatic loss of *NF2* has also been implicated in spontaneous human OSAs, schwannomas, meningiomas, mesothelioma, glioblastoma, breast, colorectal, skin, clear renal cell, prostatic and hepatic carcinomas. The Sheepdog primary lesion also had an *NFAT5-NF2* translocation which would be predicted to functionally delete *NF2*.

#### 2.3.4. Somatic deletions of genes in chromosome fragile sites were common

In addition to the genes described above, deletions with corresponding low expression were also seen in all evaluable samples in the following TSGs (COSMIC database), *ARHGEF10*, *BAP1*, *EZH2*, *FHIT*, *KMT2C*, *and STK11*. *FHIT* was deleted with ~1.1–1.3 copies in all evaluable samples. *FHIT* is located in the most common chromosome fragile site (CFS) in people, FRA3B. Gene fusions were noted in *DLG2* and *LRP1B*, in this study, which are CFSs in humans and are associated with oncogenic viral integration. These genes have not been described as fragile in the limited canine studies [27, 28]. All evaluable samples had deletions in *DMD* with ~0.5 and ~1.1 copies in the Labrador primary and Sheepdog tumors respectively. *DMD* is in a CFS, Xq21, in people and in dogs. *DMD* was previously found to be recurrently deleted in canine OSA and has been implicated as a TSG in sarcomas in people [14, 15].

#### 2.3.5. A Telomerase Maintenance Mechanism (TMM) was not identified

*TERT* was not expressed and mutation of the *TERT* promoter was not seen in any of the samples in the current study. Instead, deletion of *TERT*, the enzymatic subunit of telomerase, was seen in the Sheepdog lesions. *ATRX* gain was seen in the Labrador primary and Sheepdog metastatic lesions and moderate expression was seen in all samples. These changes are the opposite of what would be expected to give cancer cells unlimited growth potential. Telomere lengths were 0 in all tumor samples, S13 Table. The phenotypes of the tumors in this study were interpreted to be *TERT*^*negative*^*/*ALT^negative^. Regardless a basal level of elongation is thought to be required to avoid apoptosis and for proliferation. The tumors in this study may have utilized a non-defined TMM (NDTMM) or their tumors did not contain a significant number of immortalized cells and tumor heterogeneity prevented identification of a TMM. NDTMM has been described in canine melanoma and in 22% of human tumors, including glioblastoma, OSA and metastases of cutaneous melanoma [29].

#### 2.3.6. HSP90AB1, CTNNB1, CDC5L, CDH11, and MITF were amplified and expressed

Heat shock protein, *HSP90AB1*, was one of the most highly expressed genes and was amplified in the Sheepdog lesions with ~7–9 copies. *CDC5L*, a pre-mRNA splicing factor that regulates mitotic progression, was amplified in all evaluable samples with ~3–9 copies and highly expressed in the Sheepdog and moderately expressed in the Labrador. Expression of both genes was positively correlated with copy number. *CDC5L* amplification and overexpression was seen in human OSA and inhibition led to mitotic catastrophe [30]. Beta-catenin (*CTNNB1*) was another of the most highly expressed genes and was amplified in the Sheepdog lesions with ~3–4 copies. *CDH11*, a cadherin, that promotes beta-catenin nuclear localization leading to activation of the canonical Wnt pathway, was also amplified with ~3 copies in each sample and highly expressed [31]. *CDH11* overexpression is seen in invasive breast carcinomas and bone metastatic lesions but conversely high expression has been correlated with improved prognosis in human OSA [32]. *MITF* was moderately expressed in all samples and also was amplified in all samples with ~4 copies. In this chromosomal segment, *FOXP1* and *EIF4E3* were also similarly amplified. In humans, *MITF*, *FOXP1* and *EIF4E3* are also on the same chromosomal segment. An ~251 kb segment of amplification on chromosome 17 was seen in both dogs. A candidate oncogene in this region includes *ARNT*. This region also includes *CTSK*, *CTSS*, *GOLPH3L* and *HORMAD1*. The orthologous segment has been shown to be commonly amplified in human OSA [16].

#### 2.3.7. Expressed gene fusions were uncommon

All gene fusions in the primary were also found in the metastatic lesion and had low expression. The gene fusions were as follows, *GRK3-HPS4* in the Labrador and *ITGB6-CAMK2D*, *EIF4E3-MITF* and *PICALM-DLG2* in the Sheepdog, S2 Fig. The metastatic lesion in the Sheepdog additionally had 1 validated gene fusion, *MGAT5-LRP1B*. Gene fusions likely led to loss of function of these genes. In all evaluable samples there was a deletion in *DLG2*.

## 3. Discussion

A comprehensive human genome analysis pipeline (The GMS or Genome Modeling System) was successfully adapted for canine tumors [19]. This system allows for rapid, automated, and reproducible analysis of somatic alterations and sample meta-data tracking for project management. This study provides the first published data on WGS, WES and RNA-Seq in a primary and metastatic canine osteosarcoma sample from the same patient, adding to the growing genomic database in canine osteosarcoma. Comprehensive genomic analyses from the dogs in this study supports the many parallels between human and canine OSA, identifies key differences, provides evidence that the metastatic subclones have an even higher degree of heterogeneity than the primary lesion, characterizes the nature of genomic instability in canine OSA and identifies putative targetable drivers in canine OSA.

Similar to human OSA, SNVs were not common in canine OSA samples. The numbers were consistent with a similar study in pediatric OSA, where 25.2 mutations were seen per case (range 5–103) [13]. In addition, only a few of these SNVs were recurrent in canine OSA. Comparison of the SNV profiles in canine OSA studies illustrate the marked genetic heterogeneity in this disease. *NOTCH2* SNVs have been seen in many cancers, including diffuse large B cell lymphoma, marginal zone lymphoma and bladder cancer, where *NOTCH2* can function as an oncogene or a tumor suppressor gene (COSMIC, accessed 9-Jul-2019). However, only one *NOTCH2* SNV has been reported in human OSA and only one in canine OSA as far as the authors are aware [15]. Consistent with this study, the *NOTCH* SNV in the canine case was also in a metastatic lesion. *NOTCH* receptor activation in osteoblasts inhibits differentiation but is also crucial for bone development and homeostasis. *NOTCH2* also specifically enhances osteoclastogenesis [33]. *NOTCH1* and *NOTCH2* overexpression has been found in human OSA and *NOTCH1* can induce OSA in murine models [33]. In a previous canine OSA exome study, *SETD2* was the second most frequently altered gene with a frequency of SNVs of 21% and CNAs of 0% and in a subsequent canine OSA study with WGS 42% of samples had a predicted deleterious mutation in *SETD2* [14, 15]. *SETD2* point mutations were not found in our study. Nine additional statistically significantly mutated genes were identified in this previous study, *TANGO2*, *PRORSD1P*, *MAGEA9*, *LOXHD1*, *HIST1H2AH*, *F6XVW9*, *OR4D5*, *MYTIL* and *RPL18*. SNVs were not seen in these genes in the current study or in a recently published study [15]. Mutations in these genes are likely passenger mutations or common in only a subset of canine OSA cases. The low numbers of SNVs and expressed gene fusions in these two patients and in other studies would be expected to result in few neoantigens potentially limiting the efficacy of some immune therapies, although other sources of neoantigens that were not detected with the current methods are possible.

*TP53* mutations are the most common genetic alteration in human cancers, with missense SNVs being the most common mechanism (>75%) [34]. In contrast, while mutations in *TP53* have been seen in 100% of human OSA patients, most were by translocation, especially in intron 1 [13]. Human sporadic OSA is the only tumor that has been found to have a translocation in intron 1 of *TP53* [35]. This translocation was not seen in this study and has not been described in a canine tumor to date. However, *TP53* missense mutations were found in 71–83% of canine OSAs in two recent studies [14, 15]. The SNV in the Labrador sequenced here has been described before [15] while the SNV in the Sheepdog has not been described in canine OSA. The SNV in the Lab was analogous to a Y220C mutation in the human genome, which is one of the ten most common *TP53* mutations found in human cancers [36]. The SNV in the Sheepdog was analogous to the L194P mutation in the human genome, which is not one of the top fifty *TP53* mutations in human cancers but this mutation has been seen in diverse human cancers [37–39]. Both SNVs in *TP53* were in the DNA-binding domain which is the most common location for *TP53* alterations in humans and in dogs [40–43]. Mutations in the DNA-binding domain are thought to compromise the ability of this transcription factor to transactivate downstream genes that arrest the cell cycle, repair damaged DNA and induce apoptosis. Mutations thus lead to genomic and chromosomal instability. Germline loss of *TP53* in humans is known to be a risk factor for OSA [44]. *TP53* is also thought to have a protective role in mesenchymal stem cells against developing osteosarcoma [45].

Other drivers previously implicated in canine and human OSA, observed here as large-scale alterations include *TP53*, *WRN*, *PTEN*, *CDKN2A/B* and *DLG2*. *PTEN* is an inhibitor of the PI3K/Akt pathway that is crucial for cell growth and survival especially in times of stress. *PTEN* mutations are recurrent and are found in 56% of human OSAs [16, 46]. Similar to human tumors, *PTEN* deletions in both dogs were predicted to lead to permanent activation of the PI3K/Akt pathway [47]. Tumor responses to an mTOR inhibitor and pan-AKT inhibitor have been seen in a PDTX mouse model of human OSA with either *PTEN* loss or *AKT1* gain [46]. Ridaforolimus maintenance was shown to minimally but significantly increase progression free survival in adults with metastatic osteosarcomas or soft tissue sarcomas [48]. An NIH clinical trial with rapamycin therapy following standard of care treatment has recently been completed in canine OSA and the results are pending. Deletion in *CDKN2B* was seen in all samples and deletion in *RB1* was seen in the Labrador. Homozygous deletion of *CDKN2A/B* in mesenchymal stem cells was an early event in human OSA [49]. Deletion in *PTEN* and *CDKN2A* are also the most common deletions in metastatic solid tumors in people [50]. In previous studies, deletions in *CDKN2A/B* were seen in 72% of dogs and in 15–19% human OSA patients tested [14, 51–53]. Deletions in *CDKN2A/B* are also common in canine histiocytic sarcoma, 62.8%, T-cell lymphoma, 55.6%, and canine fibrosarcoma, 100% [54–56]. Germline variants in the *CDKN2A/B* loci have also been previously associated with risk of developing OSA in high-risk dog breeds [57] and also of developing histiocytic sarcoma in Bernese Mountain dogs [58]. A germline SNV may predispose the locus to a chromosomal lesion. Further, *CDK4* overexpression in this study was likely a consequence of *CDKN2B* deletion. *CDK4/6* inhibitors have been approved for estrogen receptor positive, *HER2*- metastatic breast cancers with concurrent anti-estrogen therapy and may be effective in the Sheepdog with wild-type *RB1*. *DLG2*, Disks Large Homolog 2, encodes a multi-domain scaffold protein in the Hippo signaling pathway that is important for defining epithelial polarity during cell division and loss of function of *DLG2* contributes to cell cycle progression and invasion in cancer cells [59]. *DLG2* was deleted in all evaluable samples in this study and has been shown to be one of the most highly recurrent mutations in canine and pediatric OSA and was shown to be a TSG in a cross-species study in mice, humans and dogs [59]. Mutations in *DLG2* in OSA have been exclusively via SVs.

Chromosomal instability observed here in canine OSA could be explained by the loss of cell cycle checkpoint integrity (e.g. *WRN*, *CDKN2A/B* and *RB1*), apoptotic (e.g. *FAS*) and DNA damage response pathways (e.g. *TP53*, *CHEK2* and *WRN*), SVs in CFSs and short telomeres. *WRN* encodes a DNA helicase in the RECQ family that activates the ATR CHEK1-induced S-phase checkpoint in response to ssDNA breaks and repair via homologous recombination versus NHEJ [60]. Loss of *WRN* thus leads to NHEJ mediated erroneous joining of chromosomal segments resulting in chromosomal instability and deletions [61]. Chromosome fragile sites are commonly large genes and are preferential sites for viral integration and chromosomal lesions in cancer [62]. The tumors in this study had chromosomal lesions in common human CFSs. *FHIT* is recurrently deleted in metastatic solid tumors in people [50]. Deletion of *FHIT* has been implicated as the origin of genomic instability in preneoplastic lesions via induction of replicative stress that leads to dsDNA breaks [63]. Telomere lengths measured as terminal restriction fragments (TRFs) of 12–23 kb have been estimated in canine PBMCs and mesenchymal tissues [64, 65]. Short telomeres in cells that have lost protective mechanisms (e.g. cell cycle checkpoint and p53) can lead to telomere crisis, breakage-fusion-bridges cycles and resulting chromosomal lesions. Telomere lengths in pediatric bone tumors are most commonly maintained by ALT [66, 67] and have been estimated with TRF to be 11.4 kb in primary tumors (compared to 9.7 kb in PBMCs) and 8.8. kb in the metastatic lesions [68]. In contrast 73% of canine OSAs were classified as *TERT*^*positive*^ with TRAP (Telomeric Repeat Amplification Protocol) [69]. The discordance between the TMM in canine and human OSA may be due to differences between the results from TRAP and *TERT* expression analyses or to differences in the biology of OSA between species. Telomere lengths in canine OSA have not been previously measured. The extremely low lengths seen in the OSA samples in this study could be due to a characteristic of canine OSA or alternatively individual differences, age, or inconsistencies in TRF versus the *in silico* predictor model used in this study. To determine the cause of the short telomere lengths and the absence of a TMM seen in this study, future studies specifically evaluating telomerase activity and TRF and further analysis of the accessory genes in the *TERT* and ALT pathways in canine OSA are recommended.

Chromosomal instability was most evident on chromosome 26 as it was shattered and reassembly resulted in numerous chromosomal lesions. This suggests that the method of dsDNA repair led to random chromosomal fusions. *CHEK2*, *ZNRF3* and *NF2* were deleted in both primary lesions and have been implicated in human OSA [53, 70–74]. *CHEK2* is a crucial kinase in the DNA damage checkpoint cascade that stabilizes p53 when dsDNA breaks are detected. *ZNRF3* is part of the E3 ubiquitin ligase family and inhibits cell growth and suppresses invasion and induces apoptosis via regulation of the Wnt pathway. *LZTR1*, *PTEN* and *FAS* were deleted in all lesions. Germline loss of *LZTR1*, leucine-zipper-like transcriptional regulator 1, is a cause of a type of neurofibromatosis, like *NF2* [75]. *CLTCL1* was deleted in both primary lesions. Decreased *CLTCL1*, clathrin, has been identified as an early change in breast carcinomas [76] and as a driver in oral squamous cell carcinoma [77]. *FAS* is the extracellular receptor for the death receptor pathway that leads to apoptosis or immune destruction. *FAS* is frequently downregulated in human OSA [78–80]. Localization of these TSGs onto one chromosome could contribute to the increased risk of OSA in dogs compared to humans.

Chromosomal instability likely resulted in intra and inter-individual genetic heterogeneity and led to the evolution of the clone in the primary lesion into 4 predominant subclones in the metastatic lesion of the Sheepdog. Together with the fact that more SNVs were present in the metastatic versus primary lesion, 3 possible theories may explain the dominant metastatic clone. The first and most likely is that there was selection and clonal expansion of rare tumor cells with increased metastatic potential at the primary site. The rarity of this subclone in the primary eluded detection with the methods in this study. An alternative explanation is metastasis of the clone in the primary lesion to the lungs and further mutation, which could be random, at the metastatic site, and outgrowth of clones that survived chemotherapy treatment or that had a survival advantage in the new microenvironment. The last possibility is spatial heterogeneity within the tumor. Deeper sequencing of the primary tumor, functional studies of the metastasis-specific genes, sequencing of different regions of the tumor and additional paired tumor and metastatic lesions (including sequencing of multiple metastatic lesions) would be required to determine which theory is correct.

Upregulated genes and pathways that may be targetable in the patients in this study included, *HSP90AB1*, *EP300* and the Wnt pathway. *HSP90AB1* is commonly overexpressed in cancers including human OSA [46, 81]. *HSP90AB1* functions as a chaperone to stabilize proteins during translation, and stabilize mutant, denatured or unstable proteins thus preventing their degradation. It has been considered crucial for tumor cell survival. In the Sheepdog the gene amplification and accompanying higher expression (than the Labrador) may have offered a selective advantage for the tumor cells and thus may be a driver mutation in the Sheepdog. Inhibition of *HSP90AB1* was effective in a canine OSA cell line [82] suggesting that an HSP90 inhibitor may be effective for tumors with amplification and overexpression of *HSP90AB1*. STA‐1474, an HSP90 inhibitor, has exhibited efficacy in dogs with mast cell tumors, an anti-tumor response in canine OSA cell lines and showed a partial response in 1/10 dogs and stable disease in 2/10 dogs with metastatic osteosarcoma [82–84]. *CTNNB1* is the key protein that activates the Wnt signaling pathway. Activation of the Wnt pathway leads to cell proliferation and the maintenance of stemness by turning on expression of many target genes including, *MYC*, *CCND1*, *COX2*, *S100A6*, *CD44*, *LEF1* and matrix metalloproteinases. High expression of *COX2* and *S100A6* were seen in both dogs in this study and in a previous canine OSA RNA-seq study [85]. Activation of the Wnt pathway has been implicated in many cancers including colon, breast, brain and liver cancers [86]. The Wnt pathway is also crucial for osteoblast lineage determination by mesenchymal stem cells and thus is crucial for skeletal development [87]. Overexpression of *CTNNB1* has been reported in 81% and 67% of canine primary and metastatic OSA lesions respectively and 70% of human OSA via IHC [88, 89]. Currently there are no FDA-approved drugs that specifically target the Wnt pathway but the results of early phase trials are pending [90]. *MITF* is a transcription factor that is crucial for the development and proliferation of melanocytes. *MITF* has not been implicated in OSA but is amplified, is a driver gene in human melanoma via the RAS/MEK pathway, and *MITF* inactivation leads to chemosensitivity [91]. In melanoma cell lines *MITF* amplification is commonly accompanied by *CDKN2A* inactivation and *BRAF* mutation. In this study both dogs had *MITF* amplification and the Sheepdog had a *CDKN2A* deletion and a *BRAF* amplification and thus considered as a potential driver in this study. *MITF* activity has been linked to acquired resistance to MAPK inhibitors [92] and thus it is predicted that MAPK inhibitors will be ineffective in tumors with *MITF* amplification. *MITF* is a downstream target of *EP300*, a histone acetyltransferase and an oncogene in a subset of melanomas. *EP300* was amplified in the metastatic lesion of the Sheepdog. There are no current *MITF* targeted therapies. Instead *EP300* targeted therapy have shown potential in preclinical models of *MITF* overexpressing melanomas [92]. *EP300* therapy may have been effective in the metastatic lesion of the Sheepdog with *MITF* and *EP300* amplification.

The major limitation to the current study was the small sample size, which prevented more generalized conclusions about canine and human OSA. The predicted functional consequences of the identified mutations were not confirmed in downstream analysis and would be recommended prior to clinical treatment decisions. Another limitation of this study is that both dogs had a better prognosis than the median dog with OSA and thus the genomic characteristics found in this study may not be applicable to all dogs with OSA.

## 4. Conclusions

Canine and human OSA share many genetic similarities, such as chromosomal instability leading to SVs and genetic heterogeneity between patients and to a lesser degree between the primary and metastatic lesions within a patient. Key drivers of OSA in this study that were shared with human OSA included *TP53*, *PTEN*, *CDKN2A/B*, *WRN* and *DLG2*. *CDKN2A/B* deletion is more commonly observed in canine OSA (and other canine cancers) compared to human cancers. A SNV, SV or chromatin feature in the *CDKN2A/B* loci may contribute to vulnerability to mutation in dogs. Other differences to human OSA were that a TMM was not seen in this study and *TP53* loss was by a missense mutation with accompanying LOH in dogs compared to a translocation in human OSA. Potentially actionable pathways and genes identified in this study included, *HSP90AB1*, *CDK4*, *NOTCH2*, *EP300*, mTOR and Wnt pathways. Due to the genetic heterogeneity and complexity of OSA, comprehensive genetic screening would be warranted before initiating targeted therapy in a patient. Continued efforts in comprehensive sequencing of canine OSA patients are warranted to determine if genetic heterogeneity defines subtypes in canine OSA that differ in response to treatment and prognosis and to determine how heterogeneity contributes to the development of metastatic disease.

## 5. Materials and methods

### 5.1. Samples

Tissues were obtained from the Animal Cancer Tissue Repository at Colorado State University (CSU) from client-owned dogs that were diagnosed and treated for osteosarcoma with limb amputation, chemotherapy and pulmonary metastasectomy. Informed client consent was obtained prior to tissue banking. Biobanking at the Flint Animal Cancer Center at CSU was approved by the CSU Veterinary Teaching Hospital Clinical Review Board. Matched normal tissue for each case was obtained from normal skeletal muscle. Tissues for DNA analysis were snap frozen and stored at -80°C. Tissues for RNA analysis were stored in RNAlater at 4°C.

### 5.2. Library preparation and sequencing

TruSeq stranded ribosome-depleted libraries were constructed from 500ng total RNA and single indexed. External RNA Controls Consortium (ERCC) spike-in was added to the RNA libraries (Invitrogen, 4456740). Exome capture utilized the Nimblegen Exome-Plus reagent (Nimblegen, 4000022470), covering ~152 Mb, as previously described [17]. The whole genome and exome libraries from each sample were pooled and sequenced on one lane of the Illumina HiSeqX as 2 x 151 bp reads (eWGS). The RNA libraries were pooled and sequenced on 2 lanes of the HiSeq2500 1T platform as 2 x 125 bp reads.

### 5.3. Overview of bioinformatic analysis

The McDonnell Genome Institute’s (MGI) cancer informatics pipelines, namely the Genome Modeling System (GMS), was adapted for the canine genome, S14 Table [19]. The GMS is an analysis information management system which automates all major genome analysis steps including alignment, QC, variant calling of all types, expression analysis and more, using a large number of external tools. Briefly, existing WGS/WES/RNA cancer genomic workflows originally intended to run *H*. *sapien* data were modified to work with *C*. *lupus familiaris* samples. This largely entailed replacing the reference sequence and altering/removing human specific parameters. Additionally workflows were shortened and only used for QC, alignment and variant calling. Variant annotation, CNV, and SV calling were performed ad-hoc as parts of the original workflow were not conducive to modification. We have developed publicly available versions of these pipelines, using the common workflow language and docker containers. The non-human pipelines (with options for canine analysis) are available at: https://github.com/genome/analysis-workflows/tree/master/definitions/pipelines

The workflow is outlined below and is detailed in S14 Table. Briefly, WGS and WES alignments were performed with BWA-MEM (0.7.1) [93] and duplicates were marked with Picard (1.113) (http://broadinstitute.github.io/picard/). Single nucleotide variants (SNVs) and insertions and deletions (indels) were identified with multiple callers and filtered as described below. Remaining variants were annotated with VEP (Variant Effect Predictor) using the canine reference canFam3.1 (Ensembl version 83) and retained if the observed consequence was predicted to result in a change in translation. Variants were also removed if they were found in the Dog Genome SNP Database. Differential expression analysis was performed via DESeq2 and p-value correction with the Benjamini-Hochberg procedure. Structural variants (SVs) were detected with Manta [94]. Copy number alterations (CNAs) were detected with CopyCat. All coding variants were manually reviewed. All chromosomes were analyzed except the Y chromosome and mitochondrial DNA.

### 5.4. Variant calling and filtering

Variants were identified via the somatic validation pipeline within the Genome Modeling System [19]. Briefly, SNVs were identified via SAMtools (r982) [95], VarScan2 (2.3.6) [96] and Strelka (1.0.11) [97] with identical parameters for WGS and WES samples unless otherwise noted. Similarly, insertions and deletions were identified via GATK-Somatic-Indel (5336) [98], Pindel (0.5) [99], VarScan2-Somatic and Strelka. Variant read counts were obtained via bam-readcount (0.7; -b 20 -q 20) and WES variants were removed if they met the following criteria: tumor VAF ≤ 2.5%, tumor variant reads ≤ 4, tumor coverage ≤ 10, normal VAF ≥ 10% and normal coverage ≤ 10. WGS variants were filtered in a similar manner and were removed if they met the following criteria: tumor VAF ≤ 10%, tumor VAF < = 15% in related samples, tumor variant reads ≤ 10 in WGS samples or tumor variant reads < = 15 in WES samples, tumor or normal coverage < = 20x and 40x for WGS and WES samples respectively, normal VAF > = 1% or normal variant reads > = 2 in any WGS or WES sample. Remaining variants were annotated via VEP (2.7,—pick) using Ensembl (version 83) and retained if the observed consequence were any of the following: "coding_unknown”, “essential_splice_site", “essential_splice_site, coding_unknown, intronic", “essential_splice_site, intronic", “essential_splice_site, within_non_coding_gene", “essential_splice_site, within_non_coding_gene", “frameshift_coding", “frameshift_coding, non_synonymous_coding", “frameshift_coding, splice_site", “frameshift_coding, splice_site, intronic", “non_synonymous_coding", “non_synonymous_coding, splice_site", “splice_site, intronic", “splice_site, intronic, within_non_coding_gene", “splice_site, synonymous_coding", “splice_site, within_non_coding_gene, within_non_coding_gene", “stop_gained", “stop_gained, frameshift_coding", “stop_gained, splice_site", “stop_lost", “within_mature_mirna". Variants were then removed if they were found in the Dog Genome SNP Database (DoGSD version 1; PMID: 25404132) [100]. The impact of a SNV was determined by VEP using standard criteria. Specifically, impact was determined to be high if the SNV resulted in a gain of a stop codon, frameshift or non-synonymous mutation in an essential splice site.

Additional ad-hoc filtering of SNVs included requirement of a location on chromosomes 1–38 or X, ≥7 variant reads of support and ≥4% VAF in the WGS, exome or RNA data for the tumor or metastatic lesion and ≤4 reads of support in the WGS or exome data for the normal sample. Manual review was done for variants that passed ad-hoc filtering and were genic according to a previously published SOP [21]. Variants passing filters were used to identify COSMIC signatures via deconstructSigs (1.8.0) using default settings, with the exception that trinucleotide frequencies were normalized based on their presence across CanFam3.1.

### 5.5. RNA-seq analysis

Alignments were performed with TopHat (2.0.8) and FPKM values were calculated with Cufflinks (2.1.1). Differential expression analysis was performed for tissue type (metastasis versus tumor) via the R package DEseq2 (1.14.1). Briefly Htseq (0.5.4p1) was used to obtain read counts on a gene level and genes with a cumulative read count < 1 across all samples were removed. High, moderate and low expression cut-offs were defined as follows, FPKM value in the individual sample that ranked in the top <2.5%, 2.5%-22%, and >22% for all genes respectively.

### 5.6. Structural variant and expressed fusion detection

Structural variant (SV) detection was performed with Manta [94]. Genes affected by SVs were annotated with biomaRt [101]. Ensembl gene names were recorded for the entire region affected by deletion, duplication, or inversion and for the breakpoints (plus 10kb flanking base pairs) for translocations. Fusion prediction was performed with Pizzly [102]. Gene fusions with ≥5 split or paired reads of support were considered for manual review using Svviz [103]. Gene fusions failed manual review if either gene was in a scaffold, both genes belonged to the same gene family, the second gene was adjacent to the first gene (read through), there was no SV that could explain the gene fusion, or a BLAST of reads of one gene aligned to the other fusion partner.

### 5.7. Loss of heterozygosity (LOH)

Somatic LOH was defined by first identifying a set of germline SNPs (normal bam files) with a VAF between 0.4% and 0.6% (minimum 20x coverage). The VAF for these variants was then calculated from tumor samples and plotted. Segmentation analysis was performed to identify regions of somatic LOH, defined as a mean absolute difference from baseline (0.5) of 0.1. These regions (plus 10kb flanking base pairs) were annotated with biomaRt. Germline LOH was assessed and plotted alongside somatic LOH using common canine SNP sites as regions for bam-readcount (normal bam files). The VAF was calculated from ref and variant allele counts for any SNPs with coverage ≥ 20x. Regions of germline LOH represent blind spots for somatic LOH as they contain no heterozygosity to be lost.

### 5.8. Copy-number detection

Custom annotation scripts were used to estimate mapability and gc content for the canine (canFam3.1) reference genome as described in the copyCat documentation (https://github.com/chrisamiller/copyCat). Next, the GMS tool ‘bam-windows’ was run to estimate coverage across the genome (window size = 10,000). Gap and entry_point files were obtained from the University of California Santa Cruz (UCSC) genome browser. These inputs were used with the copyCat software (https://github.com/chrisamiller/copyCat) to estimate absolute CN values and identify copy-number segments and segment values. Genes were annotated to CN segments with biomaRt.

### 5.9. Telomere length analysis

Telomere length was estimated with Telomerecat (1.0) with standard parameters [104].

### 5.10. Germline LOH

Point mutations classified as germline from VarScan2 (2.3.6) were obtained from WGS data for both samples. Mutations were then binned across chromosomes such that each bin corresponds to an approximately 500,000 base pair window and the frequency of mutations in each bin was calculated. Genomic regions where a bin exhibited a mutational frequency less than 5 were considered as LOH owing to the decrease in frequency of heterozygous single nucleotide polymorphisms (SNPs) in that area.

### 5.11. Clonal evolution

Somatic variants were obtained from WGS and WES sequencing data as described above. Variants were further filtered to SNPs and were required to meet the following criteria: (1) No normal support in either the WGS or WES data, (2) Must have WES coverage greater than or equal to 80x depth in both the tumor and metastasis samples, (3) Must have greater than or equal to 7 variant supporting reads in the tumor or metastasis samples. Variants within a genomic space that exhibited LOH or was otherwise copy-altered were removed and remaining variants passed to sciClone (1.1.0) for clustering. Resulting clusters were then manually curated and outlier variants removed. The clustered variants were then passed to clonevol (0.99.1) in order to infer clonal evolution.

### 5.12. Identification of candidate drivers

Candidate driver mutations were prioritized if a mutation of similar predicted consequence was seen in COSMIC, human OSA or other canine or human cancers, the mutation was seen in both patients in this study and/or shared in the primary and metastatic lesion. Secondarily, mutations were prioritized if consistent changes were seen in the different sequencing platforms or analysis approaches (e.g. copy number gain with high expression and vice versa, SNV with LOH).

## Supporting information

S1 Fig Filtering algorithm for SNVs located in exons. eWGS: exome + WGS. VAF: variant allele frequency.
(DOCX)

S2 Fig Few structural variants resulted in expressed gene fusions. Exon numbers are labelled. (a) *GRK3-HPS4* was caused by an inversion in the Labrador primary and metastatic lesion. A proposed structural recombination is shown. (b) *PICALM-DLG2* was caused by a deletion in the Sheepdog primary and metastatic lesion.
(DOCX)

S3 Fig Moderate correlation was seen between the SNVs in the primary compared to the metastatic lesion in the Labrador. Low tumor purity in the metastatic lesion likely decreased the correlation. VAF: variant allele frequency. If the variant was found in the WGS and exome data the highest VAF was reported. The gene names for selected VAFs were labeled.
(DOCX)

S4 Fig Four clones were seen in the metastatic lesion. Only the SNVs that were used to create the model of tumor evolution are shown.
(DOCX)

S5 Fig Kataegis was not seen in the canine OSA lesions in this study. Rainfall plot showing the intermutation distance versus genomic position for validated SNVs. Chromosomes are labelled above the figure.
(DOCX)

S6 Fig Mutational signature 1 and an unknown signature was seen in all canine OSA lesions. Signature 17 was seen in the primary and metastatic lesions in the Labrador. Signatures 9 and 15 were seen in the primary and metastatic lesions in the Sheepdog. Signatures, 6, 8, 9, 24 and 25 were seen in one lesion.
(DOCX)

S7 Fig Inter and intra-individual comparison of the genes affected by copy number alterations. (a) Amplification (b) Deletion.
(DOCX)

S8 Fig Chromosome 26 was the most affected chromosome by structural variants. SVs in the primary and metastatic lesions are shown. None of the translocations involving chr26 were found in both dogs. Only the chromosomes with a translocation involving chr26 in any lesion are shown. (a) Sheepdog (b) Labrador.
(DOCX)

S9 Fig Germline LOH was common and was extensive in the Sheepdog. (DOCX)

S10 Fig CNAs were common and genes were more likely to be affected by large deletions than other types of mutations. Summary of CNAs for all lesions are shown. Areas with copy number gains are in red and losses are in blue. Low tumor purity in the Labrador metastatic lesion precluded adequate CNA analysis of this sample. The first row is the metastatic lesion from the Labrador, the second row is the primary lesion from the Labrador, the third row is the metastatic lesion from the Sheepdog and the fourth row is the primary lesion from the Sheepdog.
(DOCX)

S11 Fig Chromosome 14 was least affected by chromosomal lesions compared to other chromosomes (normal in the Sheepdog). Representative CNA (top panel), somatic LOH (middle panel) and germline LOH (bottom panel) plots from the primary lesions in the Labrador and Sheepdog. CN = copy number; VAF = variant allele frequency. (a) Labrador (b) Sheepdog.
(DOCX)

S12 Fig Chromosomal segments with deletions commonly had LOH. A notable exception was chromosome 5 which had copy number neutral LOH. Summary of LOH for all lesions are shown. Areas with LOH (high VAF or variant allele frequency difference) are represented in a lighter color. Low tumor purity in the Labrador metastatic lesion precluded adequate LOH analysis of this sample.
(DOCX)

S13 Fig Chromosome 26 was the most affected chromosome by SVs. (a) Representative CNA (top panel), somatic LOH (middle panel) and germline LOH (bottom panel) plots from the primary lesion in the Labrador. (b) Close up view of CNAs for *PTEN* in the Sheepdog primary lesion (top panel), Sheepdog metastatic lesion (middle panel) and Labrador primary lesion (bottom panel).
(DOCX)

S14 Fig Focal biallelic deletion was seen *in CDKN2A/B*. (a) CNA (top panel), somatic LOH (middle panel) and germline LOH (bottom panel) plots from the primary lesions in the Sheepdog and Labrador. Chromosome 11 in the Sheepdog was not affected by CNA or LOH except for a focal deletion in the CDKN2 locus. (b) Close up view of CNAs in the CDKN2 locus in the Sheepdog primary lesion (top panel) and Labrador primary lesion (bottom panel). CDKN2A was not affected by a CNA or LOH in the Labrador.
(DOCX)

S1 Table Signalment and diagnostic information for the dogs that were sequenced in this study. (XLSX)

S2 Table Treatment and outcome for the dogs that were sequenced in this study. (XLSX)

S3 Table Sequencing coverage, metrics and tumor purity for each sample. (XLSX)

S4 Table (a) Samtools flagstat alignment metrics for each RNA/DNA sample. (b) Picard CollectRnaSeqMetrics alignment metrics for each RNA sample.
(XLSX)

S5 Table Manually reviewed somatic exonic small scale variants in the tumor samples sequenced are listed (synonymous SNVs are not shown). (XLSX)

S6 Table List of variants from each subclone cluster in the primary and metastatic samples from the Sheepdog. (XLSX)

S7 Table Genes affected by somatic copy number alterations in the tumor samples sequenced are listed. (XLSX)

S8 Table Somatic structural variants identified with Manta in the tumor samples sequenced are listed. (XLSX)

S9 Table Manually reviewed somatic expressed gene fusions in the tumor samples sequenced are listed. (XLSX)

S10 Table Gene expression from RNA-seq of tumor samples. Values are expressed as FPKM.
(XLSX)

S11 Table Estimation of aneuploidy in the tumor samples sequenced. (XLSX)

S12 Table Somatic loss of heterozygosity in the tumor samples sequenced. (XLSX)

S13 Table Telomere Lengths in the samples sequenced as determined by Telomerecat. (XLSX)

S14 Table Genome Modeling System pipeline. (XLSX)
