# Supplementary material for: Unraveling the chaotic genomic landscape of primary and metastatic canine appendicular osteosarcoma with current sequencing technologies and bioinformatic approaches
Source: PLoS One. 2021 Feb 8;16(2):e0246443. doi: 10.1371/journal.pone.0246443 (PMC7870011; doi:10.1371/journal.pone.0246443)
Supplement: S1 Fig — eWGS: exome + WGS. VAF: variant allele frequency. (DOCX) [file pone.0246443.s001.docx]

**S1 Fig.** Filtering algorithm for SNVs located in exons. eWGS: exome + WGS. VAF: variant allele frequency.
