# Supplementary material for: Unraveling the chaotic genomic landscape of primary and metastatic canine appendicular osteosarcoma with current sequencing technologies and bioinformatic approaches
Source: PLoS One. 2021 Feb 8;16(2):e0246443. doi: 10.1371/journal.pone.0246443 (PMC7870011; doi:10.1371/journal.pone.0246443)
Supplement: S2 Fig — Exon numbers are labelled. (a) GRK3-HPS4 was caused by an inversion in the Labrador primary and metastatic lesion. A proposed structural recombination is shown. (b) PICALM-DLG2 was caused by a deletion in the Sheepdog primary and metastatic lesion. (DOCX) [file pone.0246443.s002.docx]

**S2 Fig.** Few structural variants resulted in expressed gene fusions. Exon numbers are labelled. (a) *GRK3-HPS4* was caused by an inversion in the Labrador primary and metastatic lesion. A proposed structural recombination is shown. (b) *PICALM-DLG2* was caused by a deletion in the Sheepdog primary and metastatic lesion.

***GRK3 (****ENSCAFT00000018482.3****)***

**8**

***Chr26:19,462,733***

***(a) GRK3-HPS4***

**8**

**7**

**6**

**5**

**6**

**5**

**7**

**11**

**7**

**12**

**7**

**13**

**1**

**12**

***HPS4 (****ENSCAFT00000018633.3****)***

***Chr26:20,135,284-20,132,909***

**1**

**1**

**6**

**8**

**7**

***PICALM (***ENSCAFT00000044406.1***)***

**8**

***Chr21:13,517,652***

***(b) PICALM-DLG2***

**8**

**7**

**6**

**5**

**15**

**14**

**7**

**1**

**7**

**2**

**7**

**3**

**1**

**2**

***DLG2 (***ENSCAFT00000007339.3***)***

***Chr21:14,833,163***

**1**

**1**

**15**

**8**

**16**

Genes Deleted

CCDC83|SYTL2|CCDC89|CREBZF|TMEM126A|TMEM126B
