# Supplementary material for: Unraveling the chaotic genomic landscape of primary and metastatic canine appendicular osteosarcoma with current sequencing technologies and bioinformatic approaches
Source: PLoS One. 2021 Feb 8;16(2):e0246443. doi: 10.1371/journal.pone.0246443 (PMC7870011; doi:10.1371/journal.pone.0246443)
Supplement: S3 Fig — Low tumor purity in the metastatic lesion likely decreased the correlation. VAF: variant allele frequency. If the variant was found in the WGS and exome data the highest VAF was reported. The gene names for selected VAFs were labeled. (DOCX) [file pone.0246443.s003.docx]

**S3 Fig.** Moderate correlation was seen between the SNVs in the primary compared to the metastatic lesion in the Labrador. Low tumor purity in the metastatic lesion likely decreased the correlation. VAF: variant allele frequency. If the variant was found in the WGS and exome data the highest VAF was reported. The gene names for selected VAFs were labeled.
