# Supplementary material for: Unraveling the chaotic genomic landscape of primary and metastatic canine appendicular osteosarcoma with current sequencing technologies and bioinformatic approaches
Source: PLoS One. 2021 Feb 8;16(2):e0246443. doi: 10.1371/journal.pone.0246443 (PMC7870011; doi:10.1371/journal.pone.0246443)
Supplement: S4 Fig — Only the SNVs that were used to create the model of tumor evolution are shown. (DOCX) [file pone.0246443.s004.docx]

**S4 Fig.** Four clones were seen in the metastatic lesion. Only the SNVs that were used to create the model of tumor evolution are shown.
