# Supplementary material for: Unraveling the chaotic genomic landscape of primary and metastatic canine appendicular osteosarcoma with current sequencing technologies and bioinformatic approaches
Source: PLoS One. 2021 Feb 8;16(2):e0246443. doi: 10.1371/journal.pone.0246443 (PMC7870011; doi:10.1371/journal.pone.0246443)
Supplement: S5 Fig — Rainfall plot showing the intermutation distance versus genomic position for validated SNVs. Chromosomes are labelled above the figure. (DOCX) [file pone.0246443.s005.docx]

**S5 Fig.** Kataegis was not seen in the canine OSA lesions in this study. Rainfall plot showing the intermutation distance versus genomic position for validated SNVs. Chromosomes are labelled above the figure.
