# Supplementary material for: Unraveling the chaotic genomic landscape of primary and metastatic canine appendicular osteosarcoma with current sequencing technologies and bioinformatic approaches
Source: PLoS One. 2021 Feb 8;16(2):e0246443. doi: 10.1371/journal.pone.0246443 (PMC7870011; doi:10.1371/journal.pone.0246443)
Supplement: S6 Fig — Signature 17 was seen in the primary and metastatic lesions in the Labrador. Signatures 9 and 15 were seen in the primary and metastatic lesions in the Sheepdog. Signatures, 6, 8, 9, 24 and 25 were seen in one lesion. (DOCX) [file pone.0246443.s006.docx]

**S6 Fig.** Mutational signature 1 and an unknown signature was seen in all canine OSA lesions. Signature 17 was seen in the primary and metastatic lesions in the Labrador. Signatures 9 and 15 were seen in the primary and metastatic lesions in the Sheepdog. Signatures, 6, 8, 9, 24 and 25 were seen in one lesion.
