# Supplementary material for: Unraveling the chaotic genomic landscape of primary and metastatic canine appendicular osteosarcoma with current sequencing technologies and bioinformatic approaches
Source: PLoS One. 2021 Feb 8;16(2):e0246443. doi: 10.1371/journal.pone.0246443 (PMC7870011; doi:10.1371/journal.pone.0246443)

**S7 Fig.** Inter and intra-individual comparison of the genes affected by copy number alterations.

(a) Amplification (b) Deletion


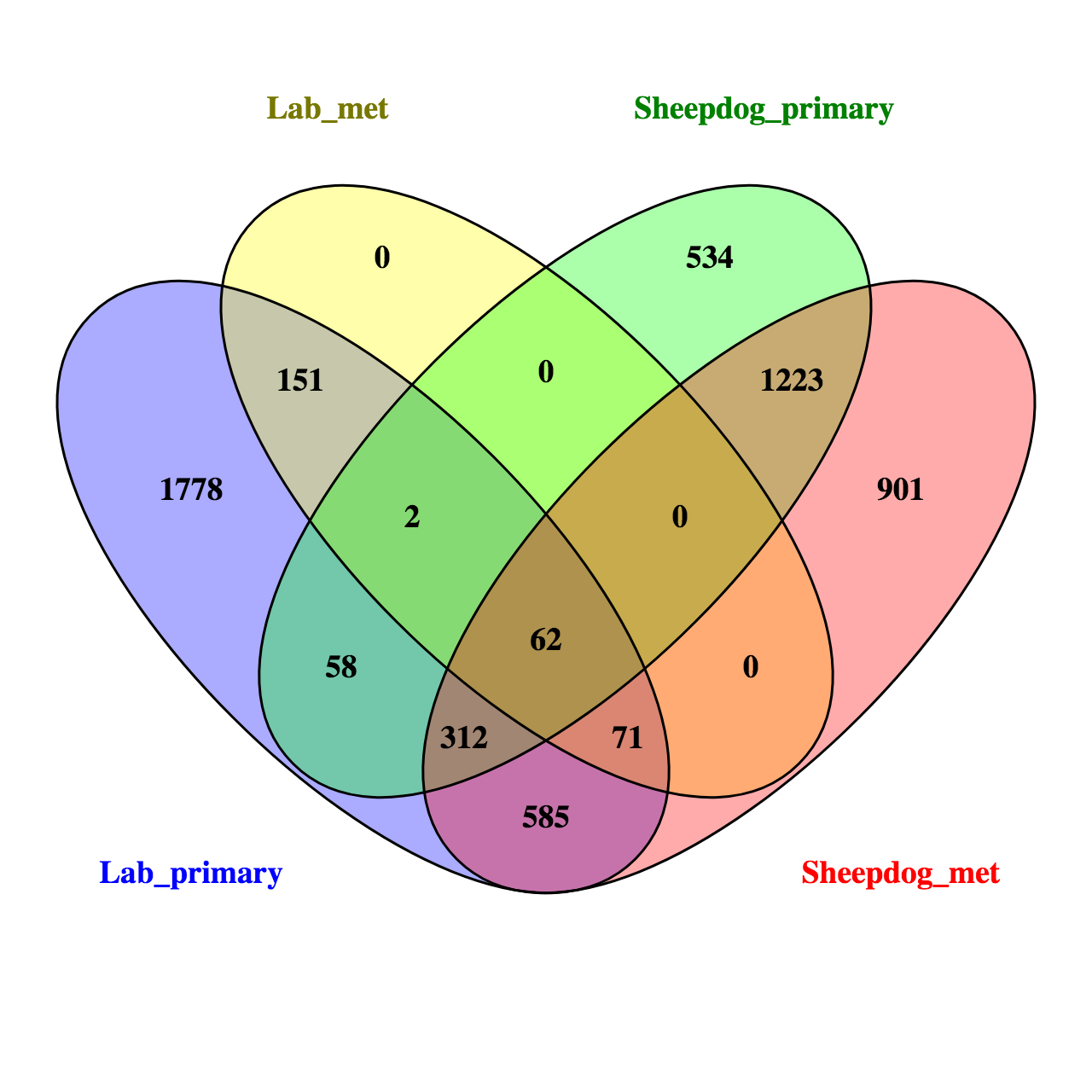

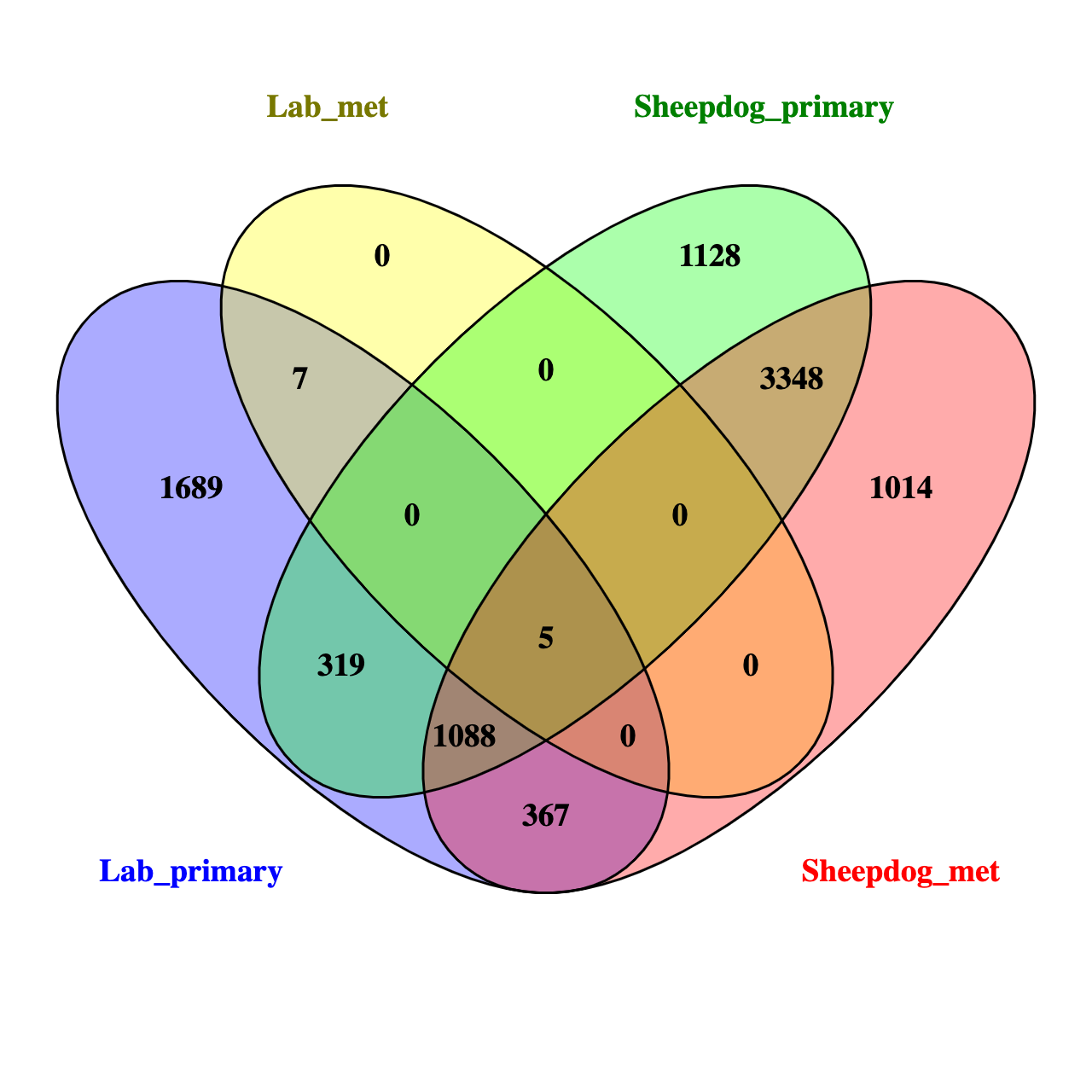

Supplement: S7 Fig — (a) Amplification (b) Deletion. (DOCX) [file pone.0246443.s007.docx]
