# Supplementary material for: Unraveling the chaotic genomic landscape of primary and metastatic canine appendicular osteosarcoma with current sequencing technologies and bioinformatic approaches
Source: PLoS One. 2021 Feb 8;16(2):e0246443. doi: 10.1371/journal.pone.0246443 (PMC7870011; doi:10.1371/journal.pone.0246443)

**S8 Fig. a and b** Chromosome 26 was the most affected chromosome by structural variants. SVs in the primary and metastatic lesions are shown. None of the translocations involving chr26 were found in both dogs. Only the chromosomes with a translocation involving chr26 in any lesion are shown.

**(a) Sheepdog**


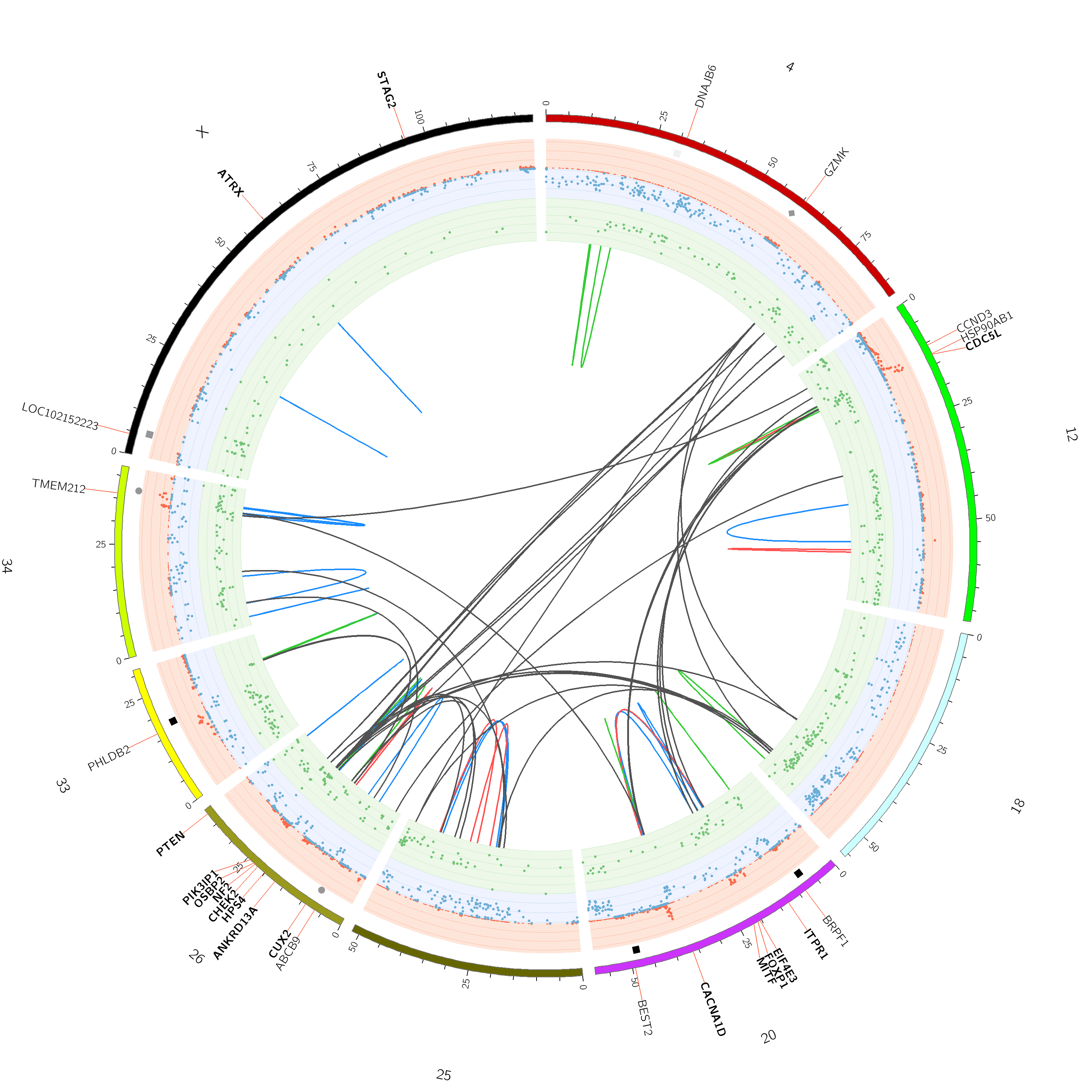


**(b) Labrador**


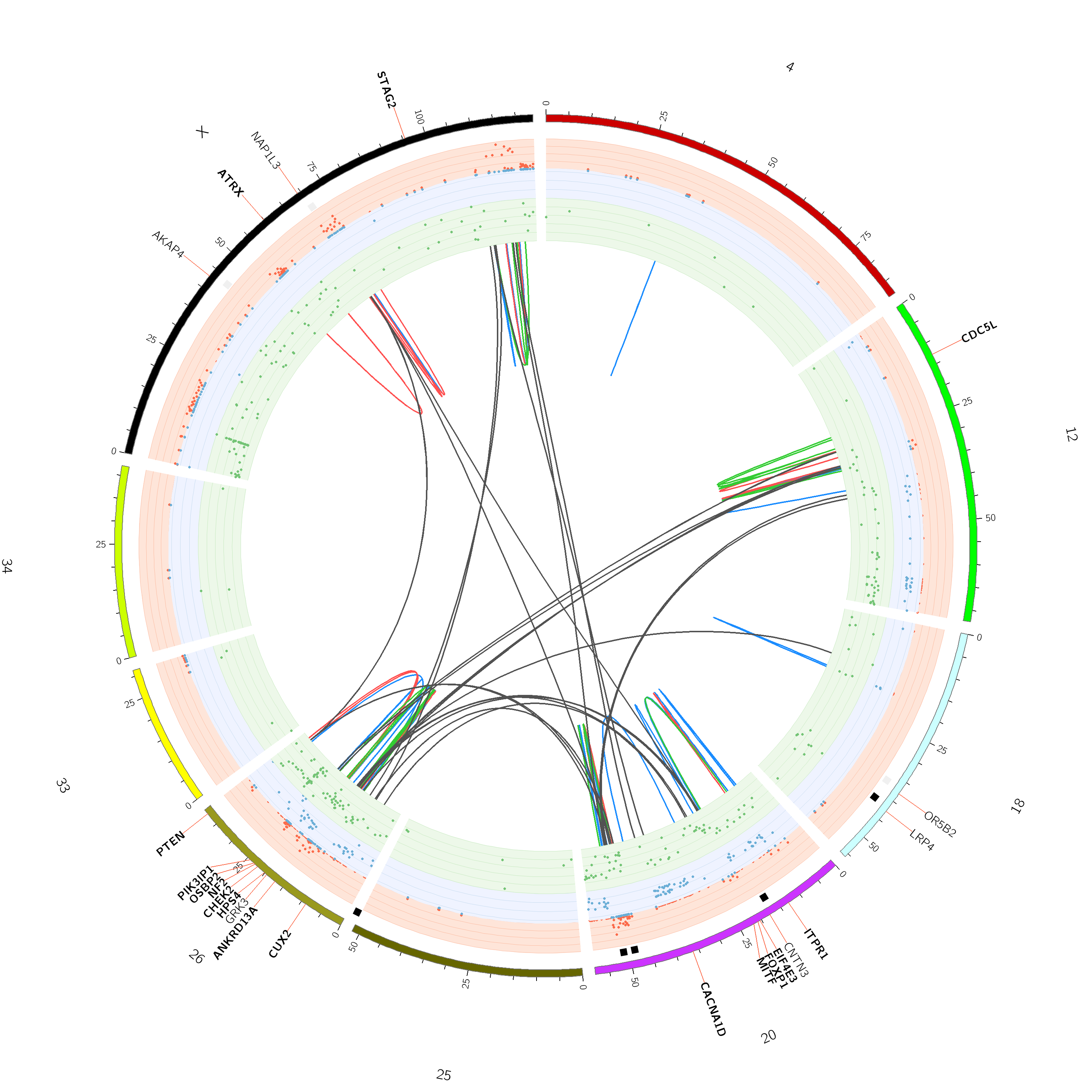

Supplement: S8 Fig — SVs in the primary and metastatic lesions are shown. None of the translocations involving chr26 were found in both dogs. Only the chromosomes with a translocation involving chr26 in any lesion are shown. (a) Sheepdog (b) Labrador. (DOCX) [file pone.0246443.s008.docx]
