# Supplementary material for: Unraveling the chaotic genomic landscape of primary and metastatic canine appendicular osteosarcoma with current sequencing technologies and bioinformatic approaches
Source: PLoS One. 2021 Feb 8;16(2):e0246443. doi: 10.1371/journal.pone.0246443 (PMC7870011; doi:10.1371/journal.pone.0246443)
Supplement: S10 Fig — Summary of CNAs for all lesions are shown. Areas with copy number gains are in red and losses are in blue. Low tumor purity in the Labrador metastatic lesion precluded adequate CNA analysis of this sample. The first row is the metastatic lesion from the Labrador, the second row is the primary lesion from the Labrador, the third row is the metastatic lesion from the Sheepdog and the fourth row is the primary lesion from the Sheepdog. (DOCX) [file pone.0246443.s010.docx]

**S10 Fig.** CNAs were common and genes were more likely to be affected by large deletions than other types of mutations. Summary of CNAs for all lesions are shown. Areas with copy number gains are in red and losses are in blue. Low tumor purity in the Labrador metastatic lesion precluded adequate CNA analysis of this sample. The first row is the metastatic lesion from the Labrador, the second row is the primary lesion from the Labrador, the third row is the metastatic lesion from the Sheepdog and the fourth row is the primary lesion from the Sheepdog.
