# Supplementary material for: Unraveling the chaotic genomic landscape of primary and metastatic canine appendicular osteosarcoma with current sequencing technologies and bioinformatic approaches
Source: PLoS One. 2021 Feb 8;16(2):e0246443. doi: 10.1371/journal.pone.0246443 (PMC7870011; doi:10.1371/journal.pone.0246443)
Supplement: S11 Fig — Representative CNA (top panel), somatic LOH (middle panel) and germline LOH (bottom panel) plots from the primary lesions in the Labrador and Sheepdog. CN = copy number; VAF = variant allele frequency. (a) Labrador (b) Sheepdog. (DOCX) [file pone.0246443.s011.docx]

**S11 Fig. a and b** Chromosome 14 was least affected by chromosomal lesions compared to other chromosomes (normal in the Sheepdog). Representative CNA (top panel), somatic LOH (middle panel) and germline LOH (bottom panel) plots from the primary lesions in the Labrador and Sheepdog. CN=copy number; VAF=variant allele frequency

**(a) Labrador**

**
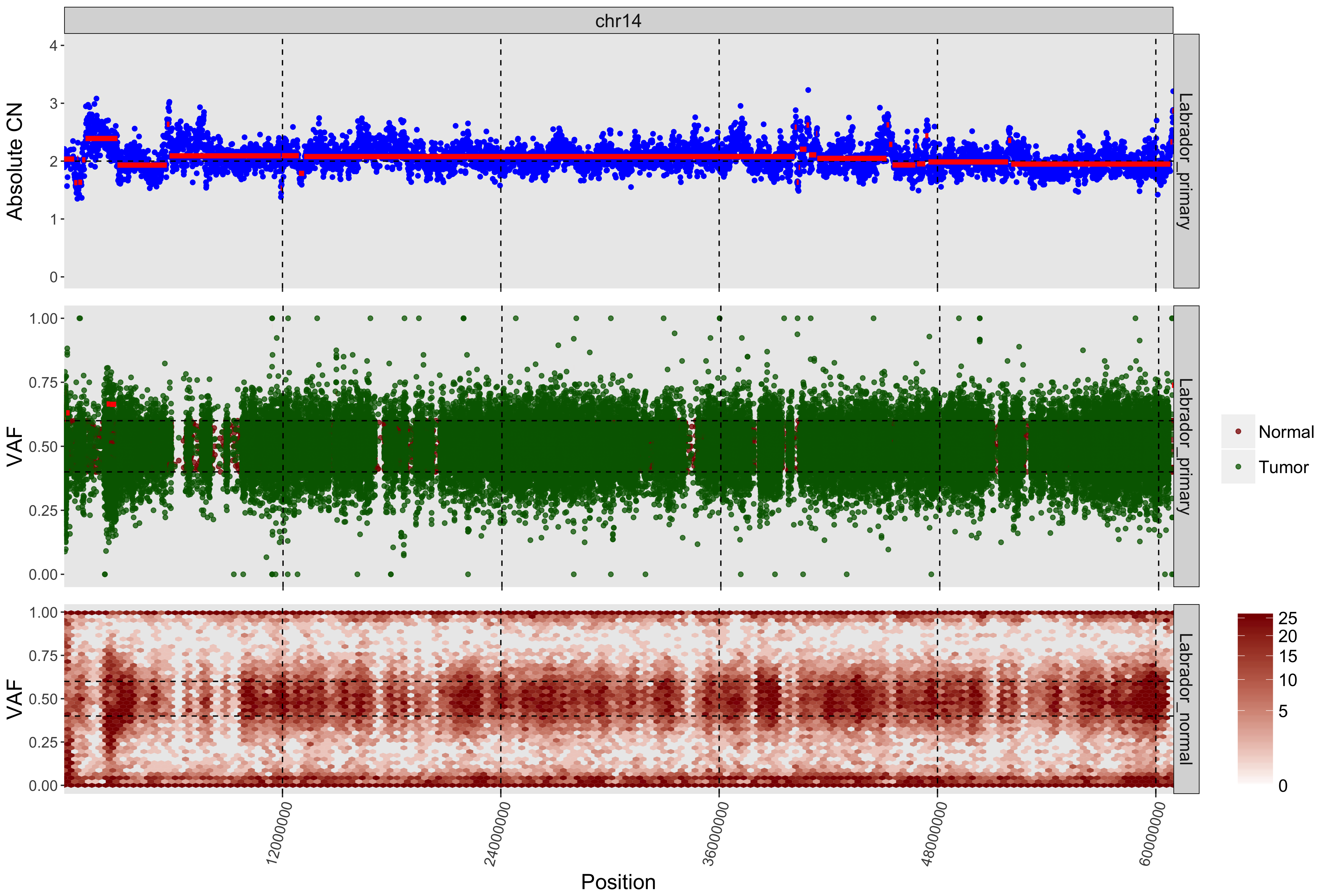
**

**(b) Sheepdog**

**
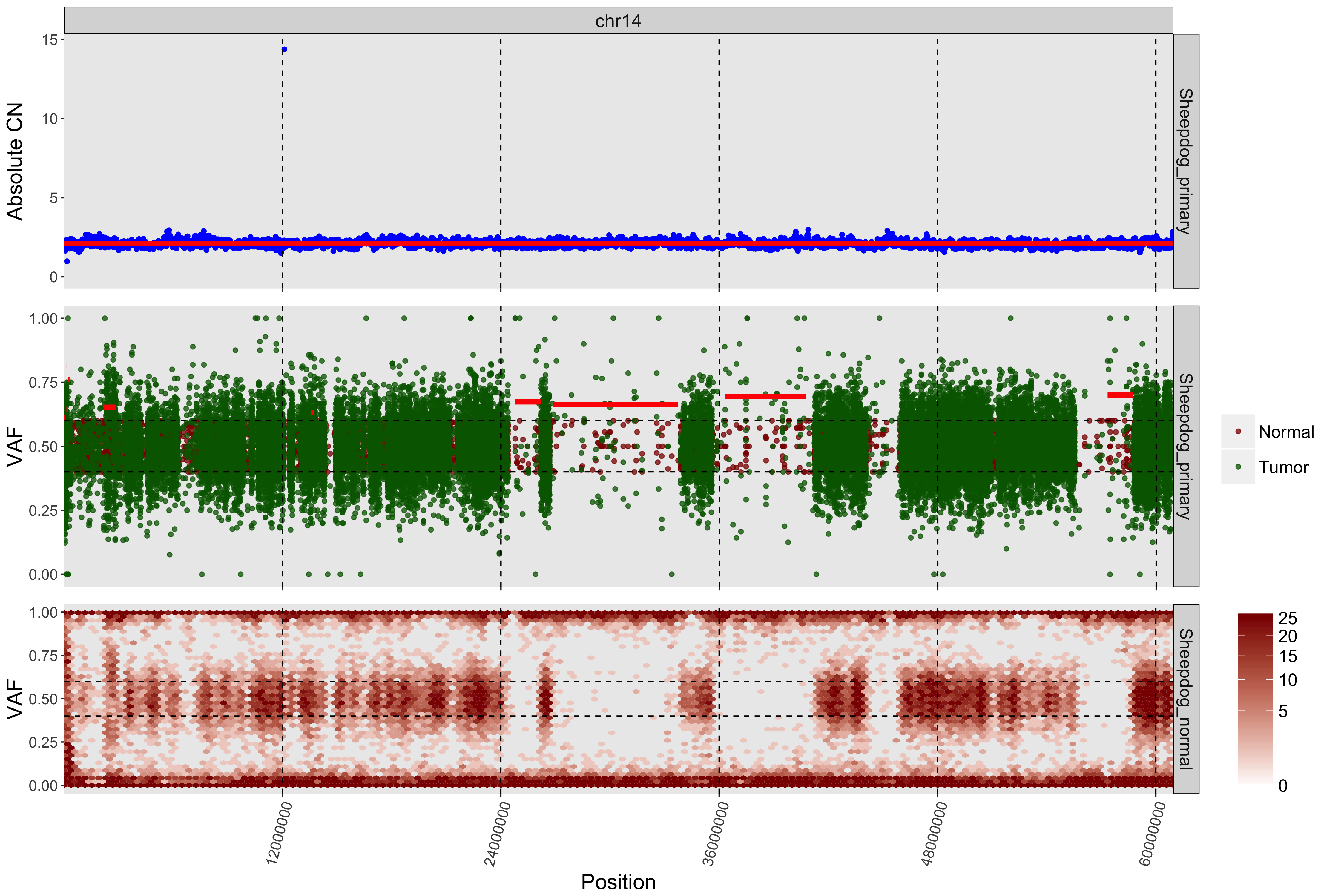
**
