# Supplementary material for: Unraveling the chaotic genomic landscape of primary and metastatic canine appendicular osteosarcoma with current sequencing technologies and bioinformatic approaches
Source: PLoS One. 2021 Feb 8;16(2):e0246443. doi: 10.1371/journal.pone.0246443 (PMC7870011; doi:10.1371/journal.pone.0246443)

**S12 Fig.** Chromosomal segments with deletions commonly had LOH. A notable exception was chromosome 5 which had copy number neutral LOH. Summary of LOH for all lesions are shown. Areas with LOH (high VAF or variant allele frequency difference) are represented in a lighter color. Low tumor purity in the Labrador metastatic lesion precluded adequate LOH analysis of this sample.


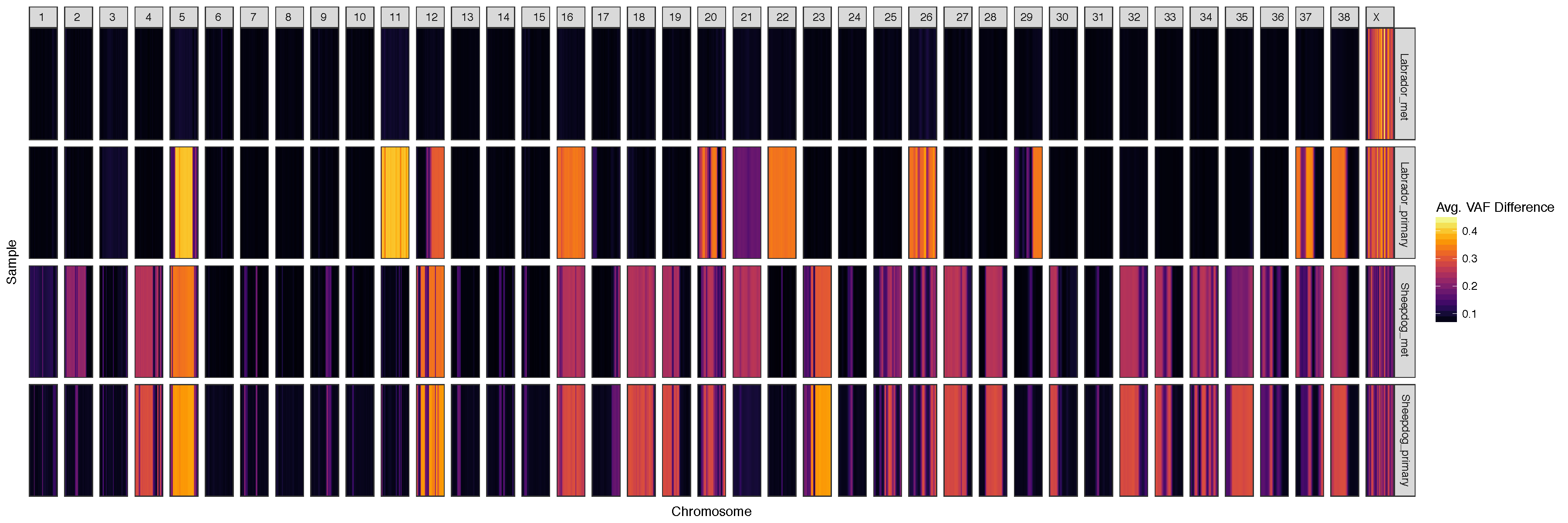

Supplement: S12 Fig — A notable exception was chromosome 5 which had copy number neutral LOH. Summary of LOH for all lesions are shown. Areas with LOH (high VAF or variant allele frequency difference) are represented in a lighter color. Low tumor purity in the Labrador metastatic lesion precluded adequate LOH analysis of this sample. (DOCX) [file pone.0246443.s012.docx]
