# Supplementary material for: Unraveling the chaotic genomic landscape of primary and metastatic canine appendicular osteosarcoma with current sequencing technologies and bioinformatic approaches
Source: PLoS One. 2021 Feb 8;16(2):e0246443. doi: 10.1371/journal.pone.0246443 (PMC7870011; doi:10.1371/journal.pone.0246443)
Supplement: S13 Fig — (a) Representative CNA (top panel), somatic LOH (middle panel) and germline LOH (bottom panel) plots from the primary lesion in the Labrador. (b) Close up view of CNAs for PTEN in the Sheepdog primary lesion (top panel), Sheepdog metastatic lesion (middle panel) and Labrador primary lesion (bottom panel). (DOCX) [file pone.0246443.s013.docx]

**S13 Fig. a and b** Chromosome 26 was the most affected chromosome by SVs.

1. Representative CNA (top panel), somatic LOH (middle panel) and germline LOH (bottom panel) plots from the primary lesion in the Labrador.


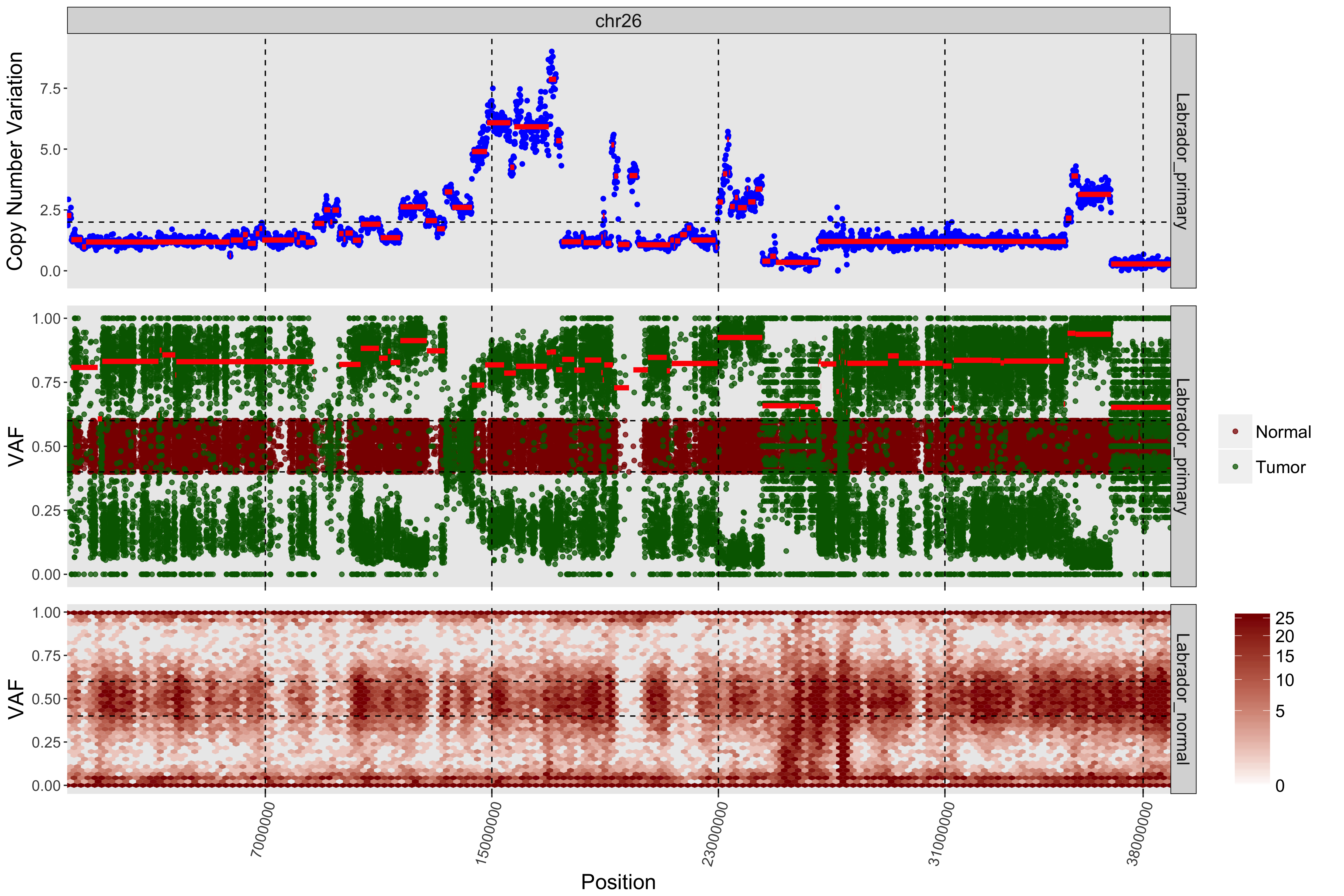


1. Close up view of CNAs for *PTEN* in the Sheepdog primary lesion (top panel), Sheepdog metastatic lesion (middle panel) and Labrador primary lesion (bottom panel)*.*
