# Supplementary material for: Unraveling the chaotic genomic landscape of primary and metastatic canine appendicular osteosarcoma with current sequencing technologies and bioinformatic approaches
Source: PLoS One. 2021 Feb 8;16(2):e0246443. doi: 10.1371/journal.pone.0246443 (PMC7870011; doi:10.1371/journal.pone.0246443)
Supplement: S14 Fig — (a) CNA (top panel), somatic LOH (middle panel) and germline LOH (bottom panel) plots from the primary lesions in the Sheepdog and Labrador. Chromosome 11 in the Sheepdog was not affected by CNA or LOH except for a focal deletion in the CDKN2 locus. (b) Close up view of CNAs in the CDKN2 locus in the Sheepdog primary lesion (top panel) and Labrador primary lesion (bottom panel). CDKN2A was not affected by a CNA or LOH in the Labrador. (DOCX) [file pone.0246443.s014.docx]

**S14 Fig. a and b** Focal biallelic deletion was seen *in CDKN2A/B*.

1. CNA (top panel), somatic LOH (middle panel) and germline LOH (bottom panel) plots from the primary lesions in the Sheepdog and Labrador. Chromosome 11 in the Sheepdog was not affected by CNA or LOH except for a focal deletion in the *CDKN2* locus.


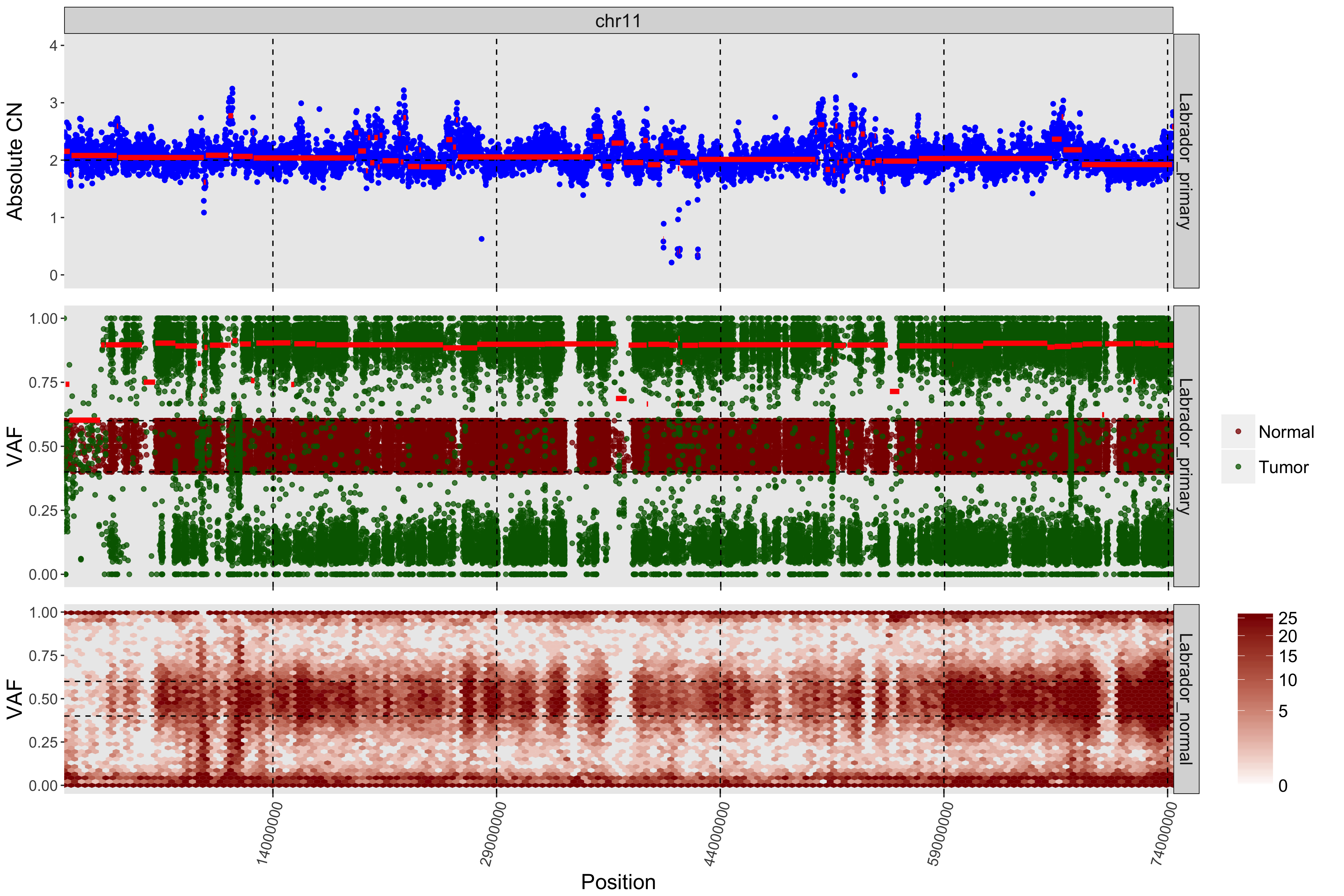

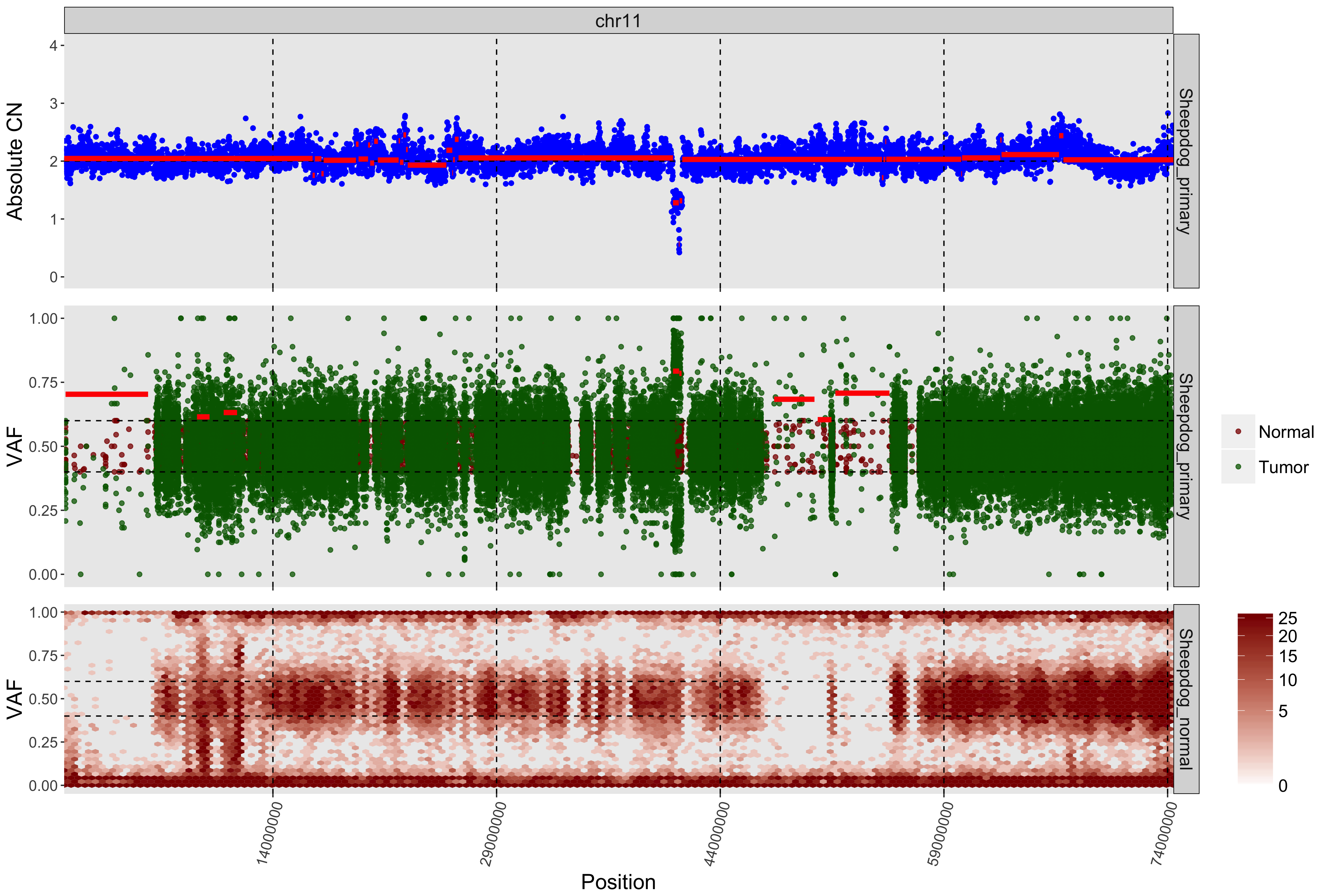


1. Close up view of CNAs in the *CDKN2* locus in the Sheepdog primary lesion (top panel) and Labrador primary lesion (bottom panel)*. CDKN2A* was not affected by a CNA or LOH in the Labrador.
